# A rapid change in magma plumbing taps porphyry copper deposit-forming magmas

**DOI:** 10.1038/s41598-022-20158-y

**Published:** 2022-10-14

**Authors:** Lawrence C. Carter, Simon R. Tapster, Ben J. Williamson, Yannick Buret, David Selby, Gavyn K. Rollinson, Ian Millar, Daniel B. Parvaz

**Affiliations:** 1 Camborne School of Mines, University of Exeter, Cornwall, TR10 9FE UK; 2 Geochronology and Tracers Facility, British Geological Survey, Keyworth, Nottingham, NG12 5GG UK; 3 Natural History Museum, Cromwell Road, London, SW7 5BD UK; 4 Department of Earth Sciences, Durham University, Durham, DH1 3LE UK; 5 Selfrag AG, Biberenzelgli 18, 3210 Kerzers, Switzerland; 6 Lightning Machines, 2nd Floor, Grove House, 774-780 Wilmslow Road, Didsbury, Greater Manchester, M20 2DR UK

**Keywords:** Economic geology, Geochemistry, Geology, Petrology, Volcanology

## Abstract

Porphyry-type deposits are a vital source of green technology metals such as copper and molybdenum. They typically form in subduction-related settings from large, long-lived magmatic systems. The most widely accepted model for their formation requires that mantle-derived magmas undergo an increase in volatiles and ore-forming constituents in mid- to lower crustal reservoirs over millions of years, however, this is mostly based on observations from shallow, sporadically exposed parts of porphyry systems. To examine this paradigm, we have evaluated the timeframe and geochemical signatures of magmatism in a ~ 8 km palaeodepth cross-section through plutonic and volcanic rocks of the classic Yerington magmatic system, Nevada. We show that the magmas in the upper parts of the system (< 8 km) underwent a major and rapid change in chemistry over a period of < 200 kyrs that is coincident with the initiation of ore formation. We attribute this change to a shift from extraction of quartz monzodiorite and quartz monzonite magmas evolving in mid-crustal reservoirs, and that had relatively poor ore-forming potential, to extraction of volatile-rich granitic magmas from greater (~ 30 km) depths. As the granites crystallised, late stage melts were intruded through the carapace as aplite dykes which contain traceable expressions of the porphyry deposit-forming fluids. The rapid nature of the shift in ore-forming potential narrows the temporal-geochemical footprint of magmas associated with porphyry mineralisation and provides new constraints for exploration models.

## Introduction

The transition to new and green technologies is increasing the need for metals^1,2^ such as copper for which demand is forecast to increase by 140–350% from 2010 to 2050^3,4^. Porphyry-type deposits provide more than 70% of global copper, around 95% of molybdenum and important amounts of gold (20%) and other metals^5^. Most form from hydrothermal fluids associated with large and long-lived calc-alkaline to slightly alkaline, water-rich and relatively oxidising trans-crustal magmatic systems, mainly in subduction-related settings e.g.^5–9^. Whilst such systems are arguably rather common, porphyry-, and particularly large porphyry-type deposits are extremely rare and increasingly difficult to find^7^. Their formation may require a series of specific conditions and events during the evolution of magmatic-hydrothermal systems.

In the drive to discover new ore deposits, there have been many recent attempts to develop whole-rock and mineral geochemical indicators to assess whether certain magmatic systems may be significantly mineralised, or ‘fertile’^10^. Their main advantage compared with conventional exploration techniques is that they are relatively cheap and are of low environmental impact. Most indicators reflect the hydrous nature of the magmas from which porphyry-type deposits form e.g.^7,11–20^.

The current paradigm is that the hydrous magmas that form porphyry-deposits result from a long (multi-million year), arc-scale, subduction-driven ‘ramp-up’ in volatiles and ore-forming constituents in mid- to lower crustal magmatic reservoirs^9,11,16,21–24^. Before emplacement into the upper crust, the magmatic system develops its ore-forming geochemical signatures over protracted time scales, in excess of ~ 5 Myrs, due to cyclical fractionation and re-charge of deep reservoirs by mafic magmas. The subduction-related tectonic regime has been suggested to progressively deepen the melt evolution zone and/or slow the upwards migration of magmas through the crust^9,22,25,26^. Alternatively, the ore-forming potential of magmas, and associated geochemical signatures, may increase during evolution within an upper crustal staging ground e.g.^5,27–29^. Distinguishing the nature of magmatic evolution in the lead up to porphyry copper ore formation is problematic because of the paucity of vertically extensive exposure over the crustal windows of porphyry ore-forming systems^30^; this has resulted in a fragmented understanding of the magmatic timescales associated with porphyry-deposit formation.

**Figure 1:**
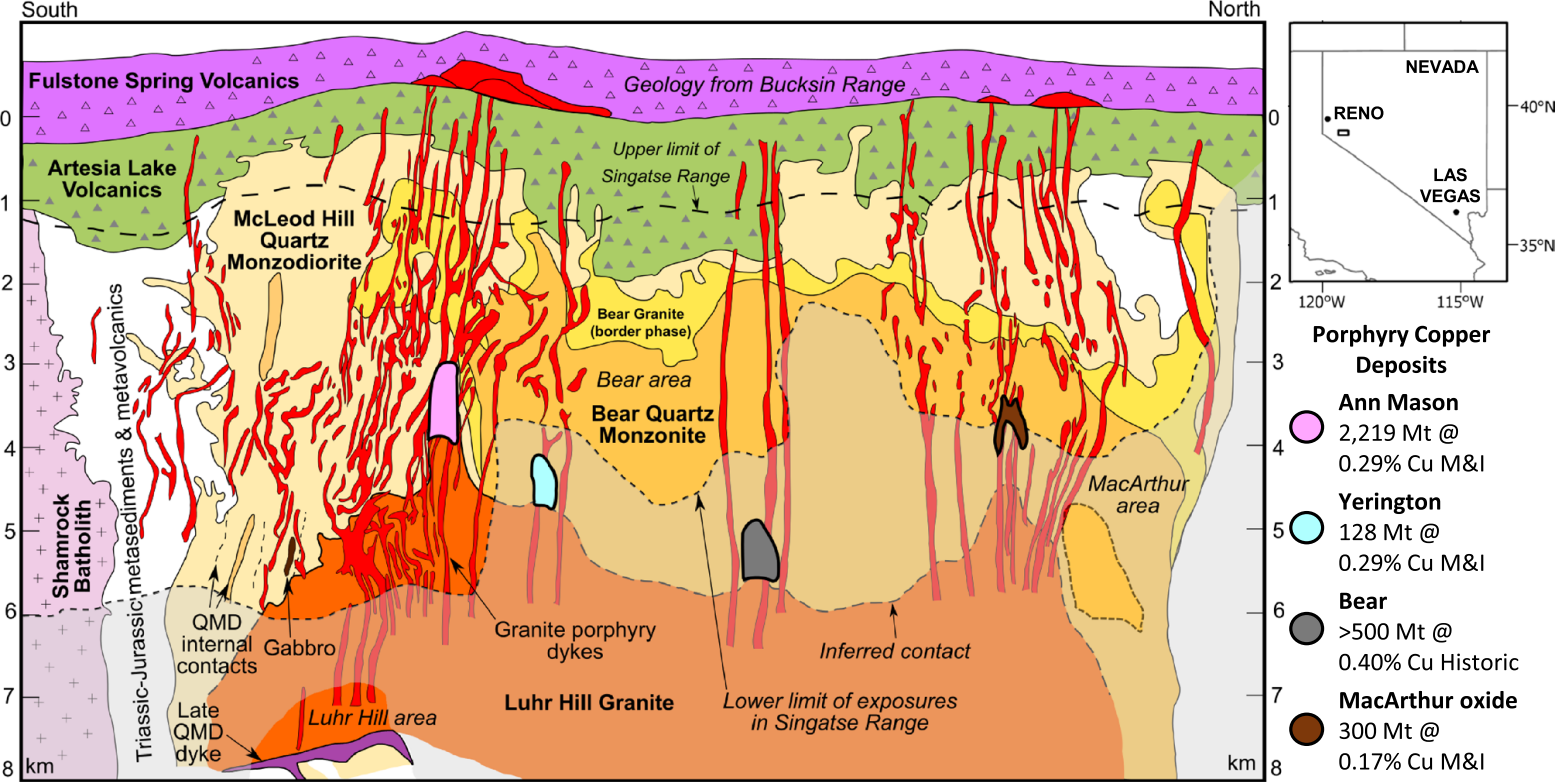
Pre-tilt cross section through the Yerington District, Nevada: Reconstructed to a palaeo-depth of 8 km, showing the intrusive units of the Jurassic Yerington batholith, the various generations of porphyry dyke swarms which were emplaced through apophyses of the Luhr Hill granite, the district’s four known porphyry copper deposits and overlying volcanics (Yerington and Bear deposits projected onto section). Section from^27,32^, with resource estimates from^42–45^. QMD = quartz monzodiorite, M&I = measured & indicated, historic = non-compliant historic estimate.

**Figure 2:**
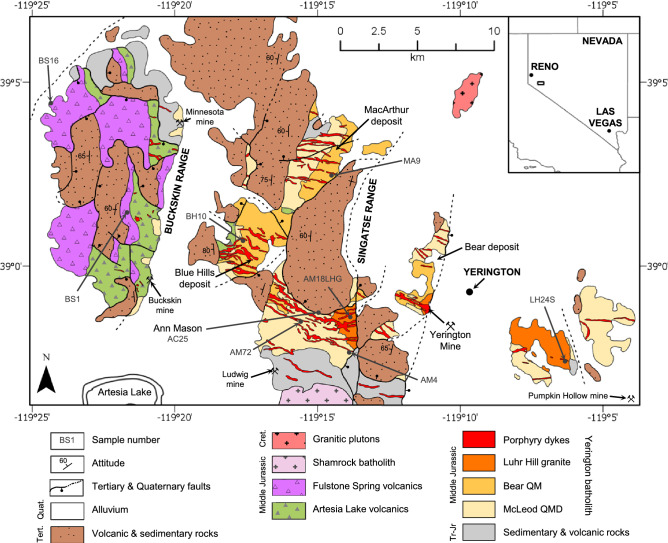
Simplified geological map of the Yerington District, Nevada: annotated with known major mineral deposits and localities sampled for zircon U–Pb CA–ID–TIMS geochronology (Fig. 4). Full sample list in Supplementary Data 1. Geology from^34,35,110^ and references therein. QM = quartz monzonite, QMD = quartz monzodiorite. WGS 1984.

To address this, the Yerington magmatic system, western Nevada, was studied as it has provided constraints for many of the most commonly used porphyry system models, mostly due to its unique ~ 8 km deep profile, from volcanic to plutonic environments, through at least four porphyry copper deposits (Figs. 1, 2) e.g.^5,6,27,28,30–33^. Here we reconstruct the Yerington magmatic system across the deep plutonic to volcanic environment, encompassing deep-seated melt evolution zones, through to the development and focusing of magmatic-hydrothermal fluids to form porphyry-type deposits. We present a new 4-D model based on the timescales and drivers for the evolution of the magmatic system’s ore-forming potential and associated geochemical signatures.

### The construction of a porphyry-forming magmatic system

The middle Jurassic composite Yerington batholith^27,34–36^ lies within a volcanic-arc terrane, in the early Mesozoic marine province^37^, which formed due to subduction tectonics to the west^35^. The batholith was emplaced into Triassic to Jurassic intermediate composition volcanics, volcaniclastic and argillaceous sedimentary rocks, and basal exposures of the likely semi co-eval Jurassic Artesia Lake Volcanics, which are unconformably overlain by the Jurassic Fulstone Spring Volcanics (subaerial quartz-latitic to dioritic lavas, domes, ignimbrites and volcaniclastics)^27,35,38^ (Figs. 1, 2). Late Cenozoic extensional faulting and associated fault block rotation in the Basin and Range has exposed a < 1 to ~ 8 km palaeodepth cross-section through the Yerington batholith^27,34,39^ (Figs. 1, 2).

There are three main plutonic phases, which, listed in order of increasing emplacement depth, are: (1) the McLeod Hill quartz monzodiorite (McLeod QMD); (2) Bear quartz monzonite (Bear QM); and (3) Luhr Hill granite (LHG)^27^. These are cross-cut by swarms of granite-composition porphyry and aplite dykes^27,33^. Units of the Fulstone Volcanics are thought to have been cogenetic with granite porphyry dykes rooted in the LHG^38,40^, or, alternatively, may have been cogenetic with the nearby younger Shamrock batholith and post-date porphyry mineralisation^35^. The dyke swarms are spatially and temporally associated with the batholith’s four known porphyry copper deposits: Ann Mason; Yerington; MacArthur and Bear (Figs. 1, 2) e.g.^27,41,42^. Combined, these host a resource in excess of 9 Mt of contained Cu^42–45^.

### Temporal constraints from field relationships

Field-based observations place constraints on the relative timing of magmatism, alteration and mineralisation. The LHG is the youngest of the three main plutons having been emplaced into the McLeod QMD and Bear QM^27^. Contacts between the LHG and previously emplaced plutons are sharp (Fig. S1), with no chilled margins or evidence of interaction with precursor granitoids. No metasomatic effects are present at the contacts beyond the later, pervasive, mostly sodic-calcic and propylitic porphyry-related alteration^46,47^. In deeper portions of the LHG (~ 7.5 km palaeo-depth, based on structural reconstructions^27^), banding is observed locally, defined by grain size variations (Fig. S2).

**Figure 3:**
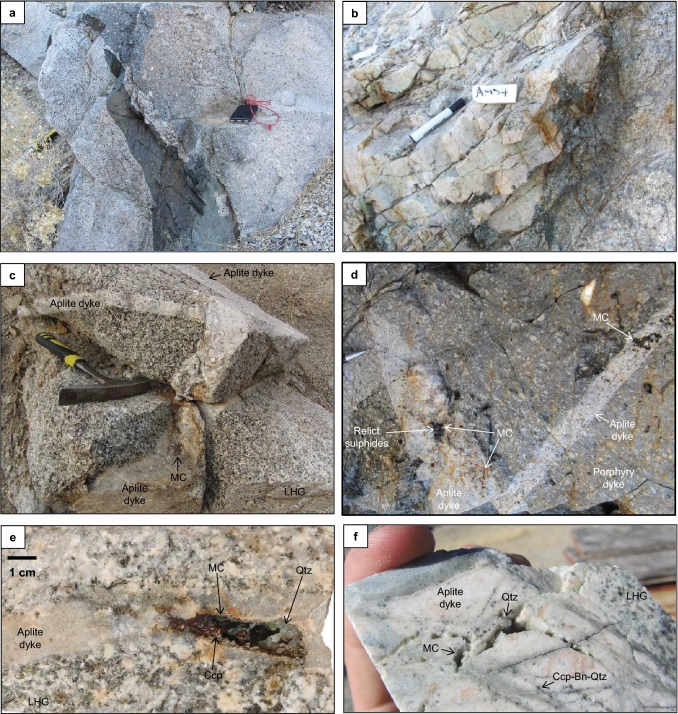
Temporal relations in the Yerington magmatic system: Field photographs of; (**a**) cross-cutting relations of multiple porphyry dyke generations which cut the LHG cupola; (**b**) lobate contacts and evidence for mingling of co-eval magmas between an aplite dyke and porphyry dyke. Secondary copper staining prevalent in the aplite dyke; (**c, d)** multiple generations of aplite dykes hosting pegmatitic segregations and mineralised miarolitic cavities (MC). The aplite dykes sharply cross-cut the cupola zone of the LHG and a porphyry dyke. Both the aplite and porphyry dykes lie palaeo-vertically beneath the Ann Mason porphyry deposit; (**e**) cupola zone of LHG cut by an aplite dyke hosting a chalcopyrite (Ccp) mineralised miarolitic cavity. This aplite dyke is sample AM13BAP in Fig. 4. Qtz = quartz; (**f**) drill core from the Ann Mason porphyry deposit showing LHG cut by an aplite dyke hosting miarolitic cavities and early chalcopyrite-bornite-quartz (Ccp-Bn-Qtz) (‘A-type’, nomenclature after^51^) veins, which locally truncate at the dyke’s margin. (**e, f**) from^33^.

The onset of porphyry mineralisation is constrained by cross-cutting relationships; it is spatially and temporally associated with multiple generations of variably mineralised granite-composition porphyry and aplite dykes that clearly cross-cut the upper (Fig. 3; S3) as well as lower parts of the LHG, and appear to have been focused through apophyses of the LHG^33,38,40–42,47,48^. The dykes generally have sharp contacts with the LHG, with some showing chilled margins and others lobate contacts (Fig. 3a)*.* It was previously suggested that both the porphyry and aplite dykes emanated from cupolas and upper zones (~ 3 to 6 km depth) of the LHG^27^, however we could not trace either to their source and therefore suggest that they were likely to have been intruded from below the deepest levels exposed in the LHG (> ~ 7 km). Different generations of aplite dykes either cross-cut and/or mingle with the porphyry dykes (Fig. 3b–d), which indicates multiple intrusion events, with some generations emplaced penecontemporaneously with porphyry dykes and others later. In the palaeo-vertically deepest (> 6 km) exposures of the LHG (or the ‘root zone’ for the porphyry deposits^30^), the majority of aplite dykes pre-date the spatially associated late-stage coarse muscovite veins and alteration^48,49^, as well as Na-Ca alteration. However, in deep exposures of the LHG, certain aplite dykes, which appear to post-date the muscovite and Na-Ca alteration, are thought to have been emplaced from a larger, longer-lived, deeper source^48^; as these post-date the hydrothermal alteration they are not considered further in this paper.

Despite the close temporal relationship between the porphyry and aplite dykes, they have very different textures. The porphyry dykes show no direct textural evidence for fluid exsolution (e.g. miarolitic cavities^50^), and are only seen to be cross-cut by mineralised veins. In contrast, certain generations of aplite dykes contain miarolitic cavities, pegmatitic segregations, early ‘A-type’ quartz ± chalcopyrite ± bornite ± molybdenite veins (nomenclature after^51^), quartz unidirectional solidification textures (USTs) which grow inwards from their margins, and are cross-cut by mineralised veins (Fig. 3c–f & S3-S6)^52^. These observations are comparable with previous descriptions of aplitic ‘vein dykes’ in other porphyry systems e.g.^53–55^. The presence of quartz USTs within the aplites is likely to indicate undercooling^56^ and rapid pressure fluctuations due to repeated carapace fracturing^55,57^ (which may induce fluid exsolution via first-type boiling^58^), suggesting that these mineralising aplite dykes were emplaced rapidly to shallow depths. Given that the aplite dykes host mineralised miarolitic cavities that are closely associated with early A-type mineralised veins (Fig. 3d–f & S4), they capture the nature and timing of magmatic-hydrothermal fluid exsolution and mineralisation. Here, we only focus on the generations of aplite dykes which cross-cut the LHG cupola and are directly associated with mineralisation.

Field relations indicate that some parts of the Fulstone volcanics were cogenetic with the emplacement of porphyry dykes associated with the LHG^38,40^. Propylitic alteration (e.g. epidote replacing primary plagioclase, and chlorite replacing mafic minerals; Fig. S7) is ubiquitous across the Fulstone Spring Volcanics, indicating that the hydrothermal system could have been active for some time after volcanism. The lack of more acid alteration (e.g. advanced argillic) may indicate that these volcanics, if related, were deposited away from the central axis of the porphyry system.

### Absolute age constraints on magmatic system evolution

**Figure 4:**
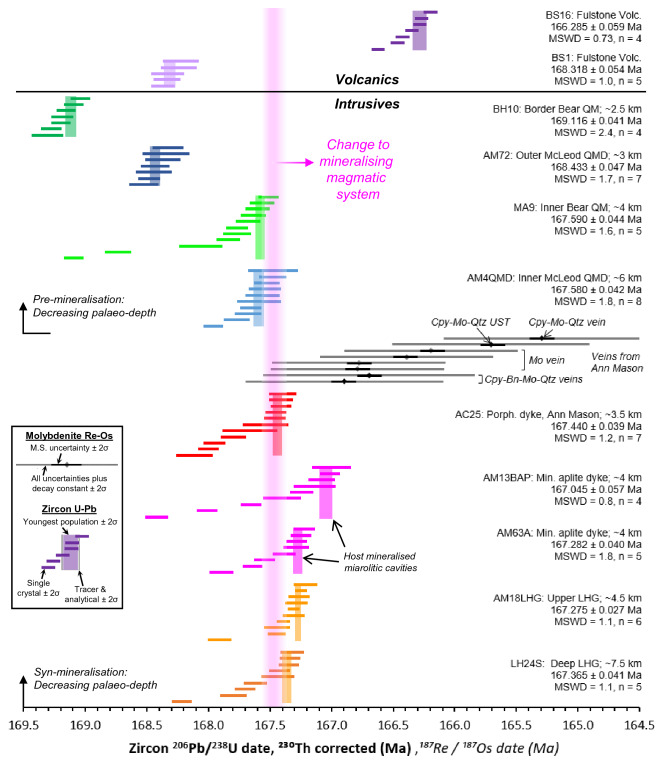
Geochronological framework for the Yerington porphyry system: Zircon single grain U–Pb CA–ID–TIMS and molybdenite Re-Os geochronological framework for samples spanning the Yerington magmatic system. Pre- and syn-mineralisation intrusive samples grouped and plotted in order of approximate palaeo-depth, after^27^. Sample details in Supplementary Data 2. We take the weighted mean of the youngest population of zircon dates that formed a statistically acceptable Mean Square Weighted Deviation (MSWD, or chi squared) as the best approximation for the crystallisation of the host magma. Cpy = chalcopyrite, Bn = bornite, Mo = molybdenite, Qtz = quartz, UST = unidirectional solidification texture, Porph. = porphyry, Min. = mineralised, M.S. = mass spectrometry. Error bars at 2σ. For comparison of Re-Os with U–Pb data, expanded uncertainties for Re-Os (typically ± 0.8 Myrs) and decay constant and tracer uncertainty for U–Pb data (typically ± 0.08 Myrs, represented in legend) should be taken into account (Refer to Supplementary Data 2).

The determination of crystallisation ages for igneous samples using U–Pb CA–ID–TIMS on zircons (See Methodology and Supplementary Data 1) provides a temporal framework for the construction of the Yerington batholith and eruption of overlying volcanics, over an indicated period of ~ 2.8 Myrs (~ 169.3 Ma to ~ 166.1 Ma; Zircon ages are reported in Fig. 4 and Supplementary Data 2. A sensitivity analysis of the ages is presented in Supplementary Data 2). This supersedes the previous U–Pb geochronological framework which was based on multi-grain TIMS^35^, and ion probe analyses^36^ on limited sample sets and which had relatively large uncertainties.

According to the new schema, the McLeod QMD pluton (AM72 and AM4QMD) was emplaced over a period of >  ~ 0.9 Myrs (~ 168.6 Ma to ~ 167.4 Ma) and the Bear QM pluton (BH10 and MA9) >  ~ 1.5 Myrs (~ 169.6 Ma to ~ 167.1 Ma). The youngest zircons for these two units are indistinguishable in crystallisation age, indicating a period of contemporaneous emplacement with crystallisation of their latest phases within ~ 100 kyrs of each other. These mineralogically and texturally distinct plutons were likely emplaced episodically to form their internal contacts^27,34^ and both appear to young downwards over a palaeo-vertical distance of ~ 3 km, supporting under-accretion e.g.^59^ as the mode of emplacement^32^.

Within the LHG, its upper region (AM18LHG; ~ 5 km palaeo-depth^27^) and a deeper portion (LH24S; ~ 7.5 km palaeo-depth^27^) show closely comparable zircon ^206^Pb/^238^U dates and weighted means of 167.365 ± 0.041 Ma and 167.275 ± 0.027 Ma, respectively (Fig. 4). These ages define the maximum emplacement time-gap between the McLeod QMD-Bear QM and LHG of 215 ± 59 kyrs. However, when the 167.440 ± 0.039 Ma age of a mineralised porphyry dyke from within the Ann Mason porphyry deposit (AC25) is considered, which cross-cuts the LHG cupola ~ 1 km higher in the system than AM18LHG, then the maximum emplacement time-gap must be shorter (140 ± 57 kyrs, or ~ 100–200 kyrs). These cross-cutting relationships imply an episodic emplacement of the exposed LHG over >  ~ 150 kyrs and the new timescale that construction of the Yerington batholith was at least two times longer than the ~ 1 Myrs previously estimated on the basis of previous geochronology^32,35^.

A stratigraphically lower unit of the Fulstone volcanics (BS1) yielded an age (168.318 ± 0.054 Ma; Fig. 4) within the emplacement duration defined by the Bear QM and McLeod QMD, whereas the stratigraphically higher unit (BS16) gave a much younger age (166.285 ± 0.059 Ma; Fig. 4), ~ 1.1 Myrs younger than the formation of the LHG cupola. This indicates the volcanic record spans over ~ 2 Myrs, rather than there having been a single post-ore volcanic event, as proposed by^35^.

From cross-cutting relations, the onset of porphyry-style Cu-Mo mineralisation in Yerington is temporally constrained by the emplacement of dyke swarms through the cupolas of the LHG e.g.^27,41,42^. In turn, our absolute U–Pb ages for the mineralised porphyry dykes that cross-cut the cupolas of the LHG, and the youngest ages for the McLeod QMD and Bear QM, both constrain the onset of ore formation to ~ 167.4 Ma (Fig. 4). The multiple generations of aplite dykes that host mineralised miarolitic cavities and early A-type veins (AM63A and AM13BAP; Fig. 3d–f & S3-S6), and have been proposed to act as conduits for the transport of mineralising fluids into the ore-forming environment^33^, capture the timing, albeit a partial record, of magmatic-hydrothermal fluid exsolution and mineralisation. As the youngest zircon growth within these aplite dykes likely crystallised as part of the magmatic assemblage at the magmatic-hydrothermal transition (See QEMSCAN, Fig. S4–S6, Supplementary Data 3), the U–Pb ages of 167.282 ± 0.040 Ma and 167.045 ± 0.057 Ma (Fig. 4) constrain the timing of mineralisation to a period of at least ~ 400 kyrs.

From Re-Os molybdenite ages for chalcopyrite-bornite-molybdenite-quartz veins (A- and B-type) (samples AC11, AC12 & AC21), a chalcopyrite-molybdenite-bearing quartz UST within an aplite dyke (or vein dyke texture e.g.^53–55^) (AC3) and a fine grained molybdenite vein (AC41MP) (Fig. 4 & S8; Supplementary Data 2) from the Ann Mason deposit, mineralisation occurred during multiple hydrothermal events over a period in excess of 1.5 Myrs, from 166.90 ± 0.1 to 165.29 ± 0.1 Ma. Comparison between the hydrothermal Re-Os molybdenite ages and magmatic zircon U–Pb ages requires that systematic uncertainties, relating to the tracer calibrations and decay constant intercalibration, must be considered, which typically equate to ± 0.8 Myrs on Re-Os dates and ~  ± 0.08 Myrs for U–Pb (Refer to Supplementary Data 2). Results therefore indicate some component of porphyry-style mineralisation within the Ann Mason deposit could potentially have occurred coincident with the eruption of the younger propylitically altered components of the Fulstone volcanics (BS16), at 166.285 ± 0.059 Ma (Fig. S7). In general, the results indicate that hydrothermal mineralisation was not a single, short-lived event.

### Geochemical change within the magmatic system

**Figure 5:**
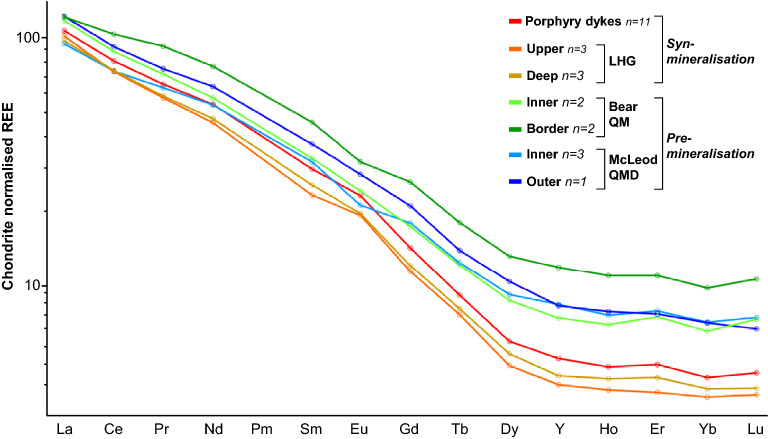
Chondrite-normalised^111^ mean whole-rock REE plots. LHG and porphyry dykes are distinct from the Bear QM and McLeod QMD intrusions, having slightly positive Eu anomalies and steeper MREE/HREE curves. All data plotted in Fig. S9.

**Figure 6:**
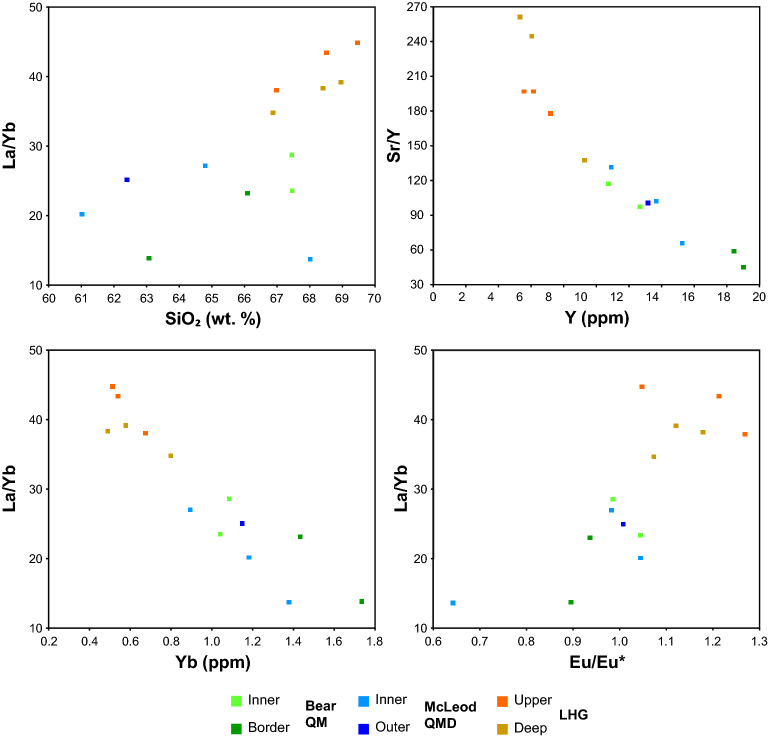
Whole-rock geochemical compositions of plutonic units in the Yerington magmatic system. Major elements in part overlap between the mineralogically distinct^27^ intrusive units. Distinct differences between pre-mineralisation (Bear QM and McLeod QMD) and inter-mineralisation (LHG) units are seen in trace element ratios. Porphyry and aplite dykes plotted in Fig. S11.

In terms of their whole-rock geochemical compositions, the McLeod QMD and Bear QM (pre-mineralisation) are similar and notably different to the LHG, porphyry and aplite dykes (syn-mineralisation) (Figs. 5, 6, S9 & S10; Supplementary Data 4). The McLeod QMD and Bear QM have similar ranges in SiO_2_ (~ 60–68 wt%) whilst the LHG samples either overlap with these or are marginally more evolved (~ 67–69 wt.% SiO_2_). The porphyry dykes show a range in SiO_2_ (60–71 wt%), whilst aplite dykes are the most evolved, generally having > 73 wt.% SiO_2_. Compared to the McLeod QMD and Bear QM, the LHG and porphyry dykes have higher Sr/Y ratios (Sr/Y > 130), steeper LREE/HREE and MREE/HREE patterns (e.g. La/Yb > 30; Gd/Yb > 3.7), lower ƩREEs (< 100 ppm), and positive Eu anomalies (Eu/Eu* > 1.05). Whilst the Dy/Yb values (~ 2) do not significantly change between the McLeod QMD, Bear QM and LHG, they follow a slightly negative trend with increasing SiO_2_ (Fig. S11).

From whole-rock geochemistry, the pre-mineralisation McLeod QMD and Bear QM are likely to be genetically related, despite their mineralogical and textural differences^27^. The more evolved composition of the syn-mineralisation LHG was probably due to a change in the bulk fractionating assemblage of the magmas. As previously shown^27^, this is likely to have been from clinopyroxene- (in which Y, MREEs and HREEs are compatible, although more weakly when compared to amphibole^60,61^) and plagioclase-dominated fractionation (in which Sr and Eu are compatible^62^) in the pre-mineralisation units, to deeper and wetter e.g.^12,13^ amphibole-dominated fractionation, with plagioclase crystallisation delayed until after emplacement into the upper crust, in the syn-mineralised units. The elevated melt-water contents led to higher Sr/Y and Eu/Eu* and depletion in HREEs^60,61,63^ (Figs. 5, 6). From the work of^64^, the slightly negative trend of Dy/Yb with increasing SiO_2_ in the plutonic units (Fig. S11) is likely to indicate that garnet did not play a role in the geochemical evolution of the system.

**Figure 7:**
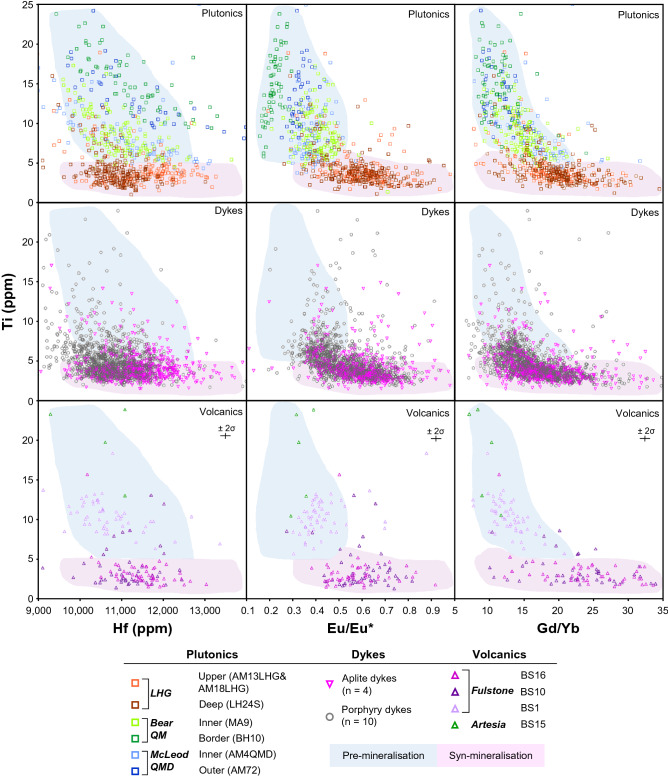
Zircon trace element signatures through the Yerington magmatic system: Zircon LA-ICP-MS trace element data from samples spanning, temporally and spatially, the Yerington magmatic system. ‘Pre-mineralisation’ and ‘syn-mineralisation’ fields shaded to aid visualisation, based on the dominant zircon geochemical signatures of the plutonic units and projected onto the plots for dykes and volcanics. Only zircon rim data have been plotted. See Fig. S13–S15 for full sample breakdown along with zircon core data.

Zircon geochemistry is a function of pressure, temperature and melt composition^65,66^ and therefore records changes in the geochemical and physical nature of the melt from its source to level of emplacement, although only during the period of zircon saturation. Zircon from across the Yerington magmatic system (Fig. 7 & S12–S15; Supplementary Data 5) can be separated into two distinct geological groups: pre-mineralisation (McLeod QMD, Bear QM and older volcanic units) and syn-mineralisation (LHG, aplite dykes and younger volcanic units). Zircon Hf concentrations, typically thought to reflect melt evolution^65^, are comparable between the pre- and syn-mineralisation units. Zircon from the pre-mineralisation McLeod QMD and Bear QM have relatively higher Ti (5–20 ppm) and lower Eu/Eu* (0.2–0.5) and Gd/Yb (MREE/HREE, 8–21) compared with the syn-mineralisation intrusives, overlapping with the pre-mineralisation Artesia volcanics and older units of the overlying Fulstone volcanics. From outer to inner portions of the McLeod QMD and Bear QM, there is an increase in zircon Gd/Yb (rising from ~ 10 to ~ 16) with decreasing Ti concentration. In contrast, zircon from the syn-mineralised LHG, aplite dykes and younger units of the Fulstone volcanics have lower Ti (2–5 ppm), higher Eu/Eu* (~ 0.4 to 0.9) and Gd/Yb (~ 10–35).

There is no major difference in zircon composition between the LHG and aplite dykes. Further, the ‘early’ and ‘late’ mineralised porphyry dykes are almost identical in their zircon compositions, from both the Ann Mason and Yerington porphyry copper deposits, in agreement with previous zircon data for dykes from the Yerington porphyry deposit^36^. Regardless of age, the porphyry dykes show no clear division between the geochemical groups of pre- and syn-mineralisation plutonic rocks, which we attribute to recycling of pre-mineralisation zircon grains from a magmatic reservoir at depth. Similarities in zircon geochemical signatures between the intrusive units and the volcanics likely indicates that are genetically linked.

As melt chemistry is largely linked to the composition and processes in the evolution zone e.g.^67^, major differences in zircon chemistry, and by extrapolation melt chemistry, between the samples is likely to reflect differences prior to magma emplacement. The comparable zircon chemistry of the McLeod QMD and Bear QM indicate that these plutons had a shared source and evolution prior to emplacement, controlled by clinopyroxene- and plagioclase-dominated fractionation, despite their mineralogical and textural differences^27^. In contrast, the syn-mineralised units (LHG and aplite dykes) underwent amphibole-dominated fractionation, evidenced by increasing MREE/HREE, and suppressed plagioclase crystallisation in the source, caused by relatively high melt-water contents, prior to significant plagioclase crystallisation post emplacement into the upper crust, the latter indicated by relatively high Eu/Eu* values^63^. Elevated Eu/Eu* in zircon may also relate to increased melt *f*O_2_ e.g.^8,29,68^, but ∆FMQ values (calculated by the method of^69^) overlap between the pre- and syn-mineralising intrusives (Fig. S16), and zircon Eu/Eu* is not a robust proxy for melt redox conditions as it is also strongly controlled by the crystallisation of other phases within the melt^70^. The low Ti concentration seen in the inter-mineralisation LHG and aplite dyke zircons could reflect lower temperatures defined by the Ti-in-zircon geothermometer^71^, induced by increased melt-water contents and reduced temperatures at which zircon dominantly crystallises within the melt^72^. However, given the paucity of good constraints on the titania activity through the evolution of the magmatic system, calculation of absolute temperatures has been avoided. Low zircon Ti concentration within later magmatic phases could also be due to decreased titania activity in the magma, due to greater incorporation of Ti into amphibole, titanite, or other Ti-bearing phases crystallising at depth. Importantly, the changes indicative of a shift from a clinopyroxene-plagioclase-dominated system to an increasingly hydrous, amphibole-dominated system at the transition from a non-mineralising to mineralising magmatic system, both suggested in previous work^27^, and whole-rock data here (Figs. 5, 6), are more pronounced within the zircon geochemistry than whole-rock geochemistry, both in the plutonic and volcanic record.

### Isotopic constraints on magma pathways

**Figure 8:**
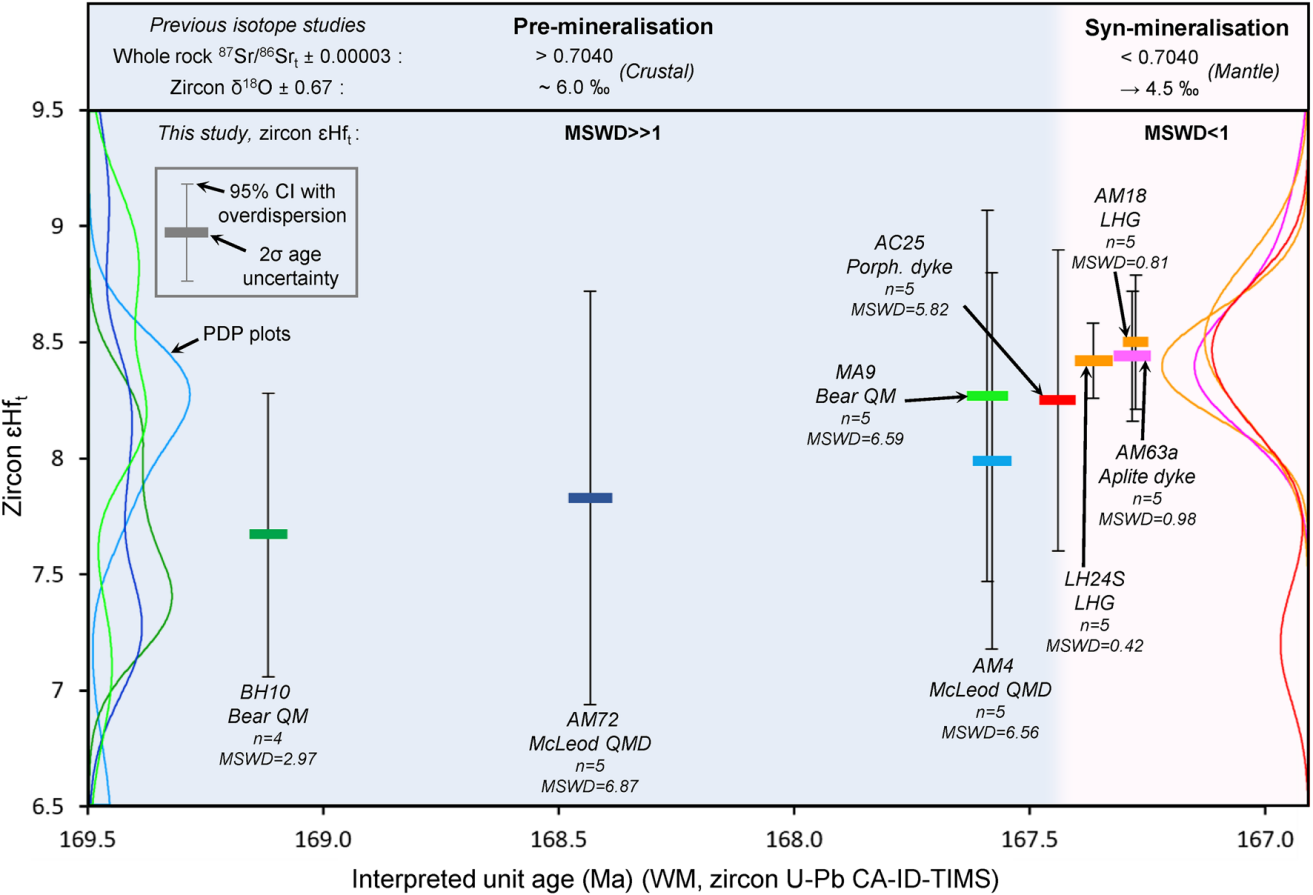
Zircon ɛHf_t_ through the Yerington magmatic system: Time corrected zircon ɛHf (ɛHf_t_) versus interpreted unit age for samples spanning the Yerington magmatic system. Age determinations for each unit are the weighted mean (WM) from zircon single grain U–Pb CA–ID–TIMS analyses (Fig. 4). ɛHf_t_ error bars at 95% confidence interval (CI) with overdispersion. ‘Pre-’ and ‘inter-mineralisation’ fields shaded. Probability density plots (PDP) inset, coloured as per sample. From the MSWD data for ɛHf_t_, ‘pre-mineralisation’ units show over dispersion (MSWD > 1) and ‘syn-mineralisation’ units show under dispersion (MWSD < 1). Data compared to previous whole rock ^87^Sr/^86^Sr_t_^27^ and zircon δ^18^O^36^ isotope studies, which show subtle shifts from crustal to mantle isotopic signatures^73–75^. All data points plotted in Fig. S17.

From whole-rock ^87^Sr/^86^Sr_t_ data^27^, there appears to have been a subtle transition from more radiogenic, crustal-like values^73^, within the pre-mineralisation intrusions, to less radiogenic ratios within the syn-mineralisation intrusions, which suggests a decreasing amount of crustal assimilation over time. Similarly, zircon O-isotopes also show a transition from δ^18^O ~ 6 ‰^36^, above values for zircon equilibrated with mantle-derived melts and indicating contamination with other crustal components, to ~ 4.5 ‰ and is within uncertainty of the expected range for the mantle^74,75^ (Fig. 8).

The ɛHf_t_ compositions of the zircon crystals that yield the youngest dates by CA–ID–TIMS U–Pb are a good approximation for the late-stage melt at the emplacement level and provide further insights into the evolution of the Yerington magmatic system (Fig. 8 & S17; Supplementary Data 6). Over the period of construction of the Yerington batholith, there were changes in the nature of zircon Lu–Hf isotopes. This is best illustrated by the weighted mean ɛHf_t_ of the sample population, and differences in the corresponding over-dispersion where the MSWD is in excess of that expected for a single population at the stated level of uncertainty. With an ɛHf_t_ uncertainty of ~  ± 0.5 ɛHf_t_ (2σ), the data show that the LHG and aplite dyke samples form reproducible single populations without over-dispersion which indicates that zircon crystallised from a melt with homogenous ɛHf, whereas the pre-mineralisation samples (> 167.4 Ma) show over dispersion that must result from variable ɛHf between zircon grains, which suggests isotopic heterogeneity within the melt. The mean value is consistent (within ~ 0.2 ɛHf_t_) between syn-mineralisation samples yet is ~ 1 ɛHf_t_ lower in the oldest Bear QM sample. The increased range and lower ɛHf_t_ indicates greater crustal assimilation, which probably occurred during ascent into the sub-volcanic environment.

Given the paucity of any major component of zircon xenocrysts older than the Triassic volcanic and sedimentary country rocks (Figs. 4, 7; Supplementary Data 2 & 5), we infer that there was little continental crustal material present to impart large variations in ɛHf_t_ upon assimilation. Nevertheless, there is a systematic variation between earlier and later pre-mineralisation intrusions that is best explained by the pre-mineralising McLeod QMD and Bear QM magmas (prior to ~ 167.4 Ma) having undergone transport, storage and evolution within, and were contaminated by, the crustal column leading to the more varied and crustal isotopic signatures (Sr, O and Hf). In contrast, after ~ 167.4 Ma, these rocks no longer show this signature, indicating no discernible crustal assimilation during magma transport and storage. It suggests that the mineralising LHG-related melts reached their evolved compositions within a lower crustal environment where they were only exposed to homogenous, mantle-derived magmas. This supports different evolution zones for the pre- and syn-mineralisation melts. These could be either discretely located throughout the crust or within the same ‘hot-zone’^67^, reflecting melt extraction in variable proximity to the country rock, with LHG melts being entirely encapsulated by juvenile, mantle-derived rocks, with negligible assimilation of other crustal components prior to emplacement.

### Depth of melt evolution

Since melt chemistry is partly controlled by the pressure of differentiation e.g.^67^, it can offer insights into the depth at which melts evolve. For example, increased Sr/Y, as observed in the LHG, is often used to infer a greater depth of fractionation due to an increased abundance of amphibole and suppressed plagioclase crystallisation within a relatively deep fractionating assemblage^12,13,67,76^. The compositions and normative mineralogy of H_2_O-saturated minima and eutectics for haplogranitic melts also share a relationship with pressure e.g.^77,78^. This pressure equates to the approximate depth at which the melt reached the eutectic, or evolved to its bulk composition, rather than the emplacement depth.

**Figure 9:**
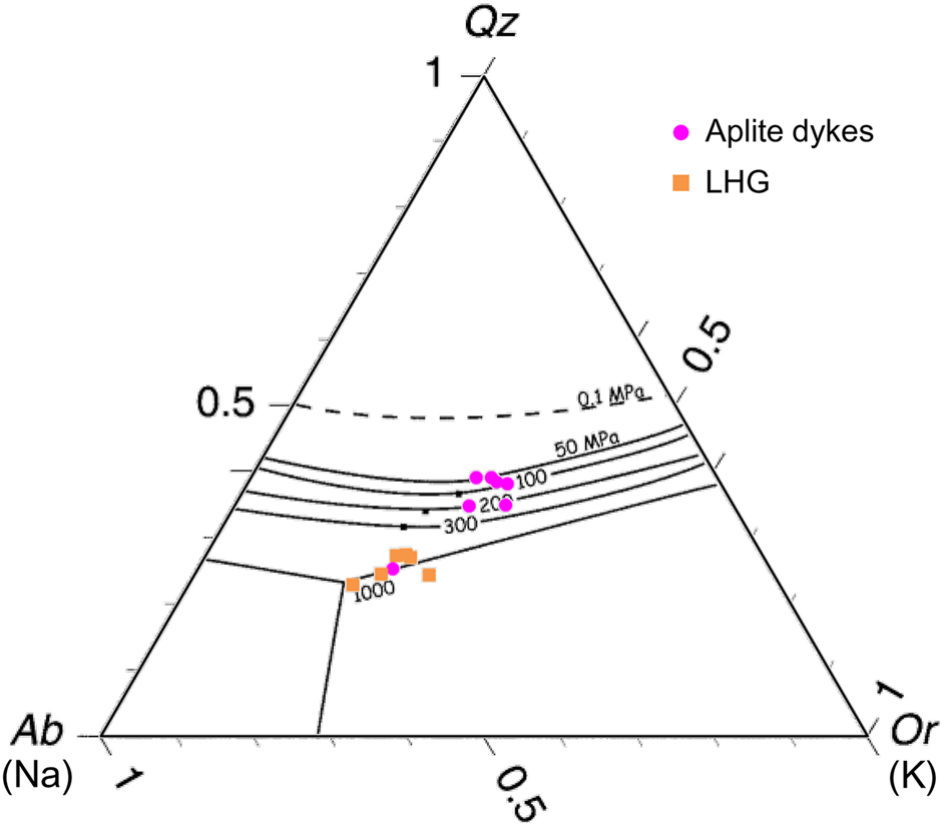
Depth of different magma sources: CIPW normative mineralogy (method of^90^) from whole-rock XRF data for aplite dykes and LHG plotted on the H_2_O-saturated haplogranitic melt minima plot of^78^ (after^77^). Samples overprinted by intensive Na–Ca alteration are not plotted. Porphyry dykes plotted in Fig. S18.

The normative mineralogy of the LHG, porphyry and aplite dykes show a close fit to the H_2_O-saturated minima and eutectics for haplogranitic melts e.g.^77,78^ (Fig. 9 & S18). LHG samples and porphyry dyke samples cluster between the ~ 450 MPa and 1000 MPa minima. Conversely, aplite dyke samples cluster between the ~ 75 MPa and 200 MPa minima. Assuming lithostatic pressure with an average overburden density of 2.5 g/cm^3^, determined pressures roughly equate to a melt evolution depth of ~ 20–40 km for the LHG and porphyry dykes, and ~ 3–8 km for aplite dykes.

As with the geochemical signatures (Figs. 5, 6, 7), which are indicative of a deeper amphibole-dominated evolution e.g.^12,13^, these melt minima relationships also support a deep melt evolution (~ 20–40 km) for the LHG, and also probably for the porphyry dykes (Fig. S18), rather than shallow fractionation at the emplacement level. However, this whole rock barometry indicates that aplite dykes (including those that contain textures indicating fluid exsolution and Cu mineralisation^33,52^; Fig. 3 & S3-S6), were sourced from shallow depths (~ 3–8 km), near their level of emplacement, probably representing late-stage melts associated with the LHG.

### A rapid change in magmatic plumbing to tap porphyry ore-forming magmas

**Figure 10:**
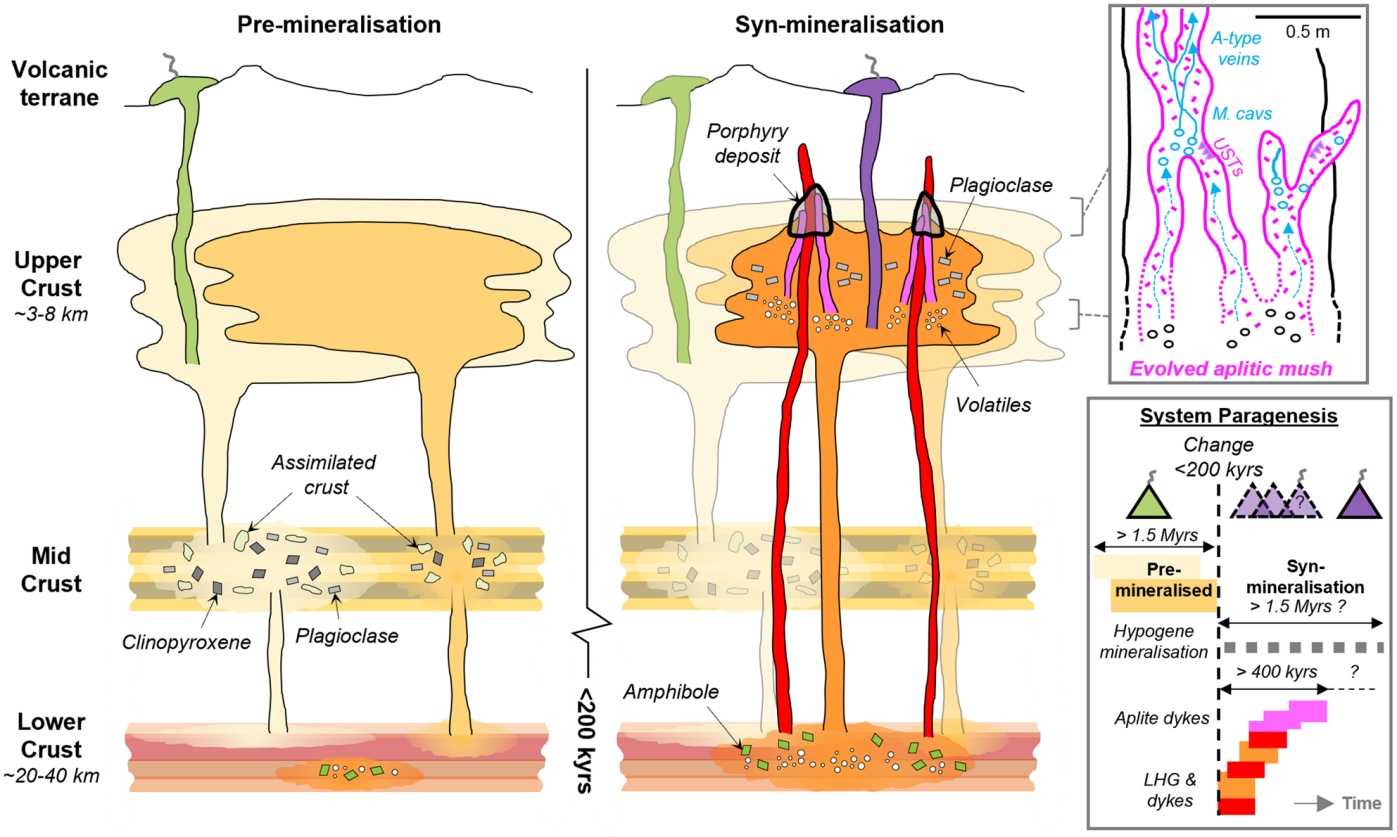
A rapid change in magmatic plumbing to tap porphyry-mineralising magmas: Simplified system paragenesis and conceptual cross-section through the porphyry deposit forming magmatic system. A long lived (> 1.5 Myrs) evolution and contemporaneous emplacement of precursor plutonics, with volcanic activity, was followed by a rapid (< 200 kyrs) change in magmatic plumbing to tap fertile porphyry deposit-forming magmas from a ~ 20–40 km deep lower crustal staging ground where they predominately underwent amphibole-dominated fractional crystallisation. From this zone of melt evolution, fertile magmas were emplaced into the shallow crust to form plutons and porphyry stocks, and underwent further differentiation at ~ 3–8 km depth, including extensive plagioclase crystallisation, with episodic upward injection of multiple generations of aplite dykes for >  ~ 400 kyrs, which likely acted as crystal mush conduits for mineralising fluids^33^. As mineralising fluids exploited these conduits, porphyry deposit formation continued episodically for potentially in excess of 1.5 Myrs, and may have been co-eval with volcanism. M. cavs = miarolitic cavities, USTs = unidirectional solidification textures. Vein nomenclature after^51^. Modified after^5,33,67^.

Previous models for the assembly of the Yerington batholith e.g.^27,32,47^ suggest sequential emplacement of the McLeod QMD and Bear QM, yet this is at odds with the revised chronology where these two intrusive units crystallised and must have been emplaced at least in part over the same ~ 800 kyrs time period (Fig. 4, 10). Whilst these units are mineralogically and texturally distinct^27^, their isotopic signatures and whole-rock and zircon geochemistry are very similar (Figs. 5, 6, 7, 8), suggesting similar sources and evolutionary pathways, likely in a mid-crustal storage zone (Fig. 10), although this must have evolved over the 1.5 Myrs of upper crustal activity. The whole-rock and zircon geochemical signatures of the pre-mineralisation McLeod QMD and Bear QM units (Fig. 5, 6, 7 & S9–S15) are consistent with clinopyroxene-plagioclase-dominated fractionation in the mid-crust (Fig. 10). This contrasts with the change to the syn-mineralisation signatures (Figs. 5, 6, 7 & S9–S15) which indicate an amphibole-dominated lower crustal evolution (~ 20–40 km depth; Figs. 9, 10). The intrusions either-side of this geochemical change are both relatively evolved and have comparable indicators of fractionation, such as whole-rock SiO_2_ and zircon Hf concentrations (Figs. 6, 7).

The shift in the compositions of the magmas which formed the pre-mineralising intrusions and then the LHG, along with the change in the dominant fractionating assemblage, is constrained to within < 200 kyrs and is coincident with the onset of porphyry mineralisation. For the LHG and porphyry dykes, although there is little difference in their whole-rock (Fig. 5 & S10), zircon trace element and isotopic compositions (Fig. 7, 8), and where they plot on the melt minima diagram (Fig. S18), there is no evidence from within the ~ 8 km depth of exposure that the porphyry dykes were derived from the upper parts of the LHG (as per previous models e.g.^27^). Instead, the porphyry dykes may reflect the same or similar intrusive events that formed the LHG. Once emplaced at shallow crustal levels, the LHG magmas underwent further fractionation (at ~ 3–8 km depth, based on melt minima plots; Fig. 9), potentially forming igneous banding textures (Fig. S2), to form more evolved and volatile-rich melts that were episodically injected as aplite dykes over a period of at least ~ 400 kyrs (Fig. 4). Multiple, episodically emplaced generations of these aplite dykes, which provide textural evidence for undercooling and exsolution of mineralising fluids (mineralised miarolitic cavities^33,52^), are associated with early A-type veins and likely acted as crystal mush conduits for mineralising fluids^33^ (Fig. 3c–f & S3–S6). Zircon U–Pb and molybdenite Re-Os ages indicate hydrothermal mineralisation occurred episodically over time-periods potentially in excess of 1.5 Myrs post-emplacement of the LHG cupola (Fig. 4), perhaps coincident with the eruption of the younger propylitically-altered components of the Fulstone Volcanics that bear the same zircon geochemical signatures as the LHG (Figs. 4, 7, S7, S12-S15). The proposed time-period for porphyry ore formation, which may have exceeded 1.5 Myrs post emplacement of the LHG cupola, is not uncommon for medium to large scale, composite porphyry systems e.g.^9,79–82^, or where multiple, episodic ore-forming magmatic-hydrothermal events are documented e.g.^72,81,83^. This all suggests that relatively evolved, internal or deep parts of the LHG remained active and continued to produce the magmas and associated magmatic-hydrothermal fluids responsible for porphyry deposit formation after emplacement and crystallisation of the cupolas and upper regions of the LHG (Fig. 10). It also implies that the LHG pluton was episodically recharged rather than being emplaced as a single intrusive event, as previously suggested^27,38^.

Given the similarities in zircon trace element geochemistry between the mineralised porphyry dykes from the Ann Mason and Yerington porphyry deposits (Figs. S13–S15;^36^), and their mineralogy^27^, they are probably genetically related. By extrapolation, this is also likely to be the case for porphyry dykes in the Yerington Districts’ two other known porphyry deposits: Bear and MacArthur. Because of this, it is probably salient for future computational simulations of batholith construction and mineralisation to include fluids derived from across all porphyry centres; this will yield a considerably larger copper endowment than when individual porphyry centres are considered (> 9 Mt of contained Cu^42–45^).

The abrupt (~ 100–200 kyrs) change to geochemical signatures indicative of magmas from a lower crustal amphibole-stable, plagioclase-supressed, evolution zone (from whole-rock Sr/Y and REE patterns, and zircon geochemistry, Figs. 5, 6, 7), in tandem with an increase in ore-forming potential, requires an explanation. It is plausible that these changes could have occurred in a single magma reservoir as a result of a progressive long-term transition. Within the lower crust, the rapid change in magma chemistry could reflect a relatively discrete temporal point at which the ‘amphibole-in’ line was suddenly crossed. This could occur either due to a build-up of volatiles following fractionation of anhydrous phases, over a period of at least 1.5 Myrs (Fig. 4), or because of an injection of new melts into a lower crustal clinopyroxene cumulate pile or ‘sponge’ that reacts with new melt to become progressively replaced by amphibole^76^. The dated porphyry dyke that sits at the temporal onset of mineralisation (AC25; Fig. 4), and has zircon geochemistry appearing to ‘straddle’ the pre-and syn-mineralisation signatures, could mark this threshold being crossed in a transitional phase of magmatism, although this is a feature common to all porphyry dykes, regardless of their timing (Fig. 7 & S13–S15).

Although we cannot rule out a model where the change captures a single petrological event in a transitional process, there are several features that do not support progression within a single magma evolution zone. If the change were merely due to a transition in the magma supplied to the upper crust we would perhaps not expect the sharp contacts between the mineralogically distinct McLeod QMD and LHG plutons^27^. A scenario where the fractionating assemblage suddenly changes in a single transitional melt extraction zone is also challenging to reconcile given the variations observed in the isotopic data (Fig. 8), i.e. from a heterogeneous distribution, indicative of variable interaction with crustal components, to a homogeneous, less contaminated, mantle-derived signature within < 200 kyrs. Instead, magmas being sourced from discrete melt evolution zones within the crust, with pre-mineralisation intrusions evolving at shallower levels, surrounded by country rocks, and the ore-related intrusions evolving within a deeper zone dominated by mantle-derived rocks would be a better fit to the data (Fig. 10). This idea is also supported by the geochemical indicators of melt evolution depth (Figs. 5, 6, 7, 9 & S18). We envisage that the earlier, pre-mineralisation stage magmas were derived from the mid-crust. During protracted storage and evolution, these assimilated crustal materials. The magmatic plumbing then shifted to tap magmas from a deeper, lower crustal ‘hot-zone’ (~ 20–40 km; Fig. 9, 10)^67^, which likely evolved over extended time periods. In this scenario, the pre-mineralisation geochemical signature of the zircon cargo of the porphyry dykes would be acquired as they intruded up through the pre-cursor magmatic system on route to their level of emplacement. It is also feasible that the mid-crustal melt evolution zones of the McLeod QMD and Bear QM could have remained active post emplacement, or during the evolution of the syn-mineralisation magmas. In addition, the geochemical differences do not exclude progressively more oxidising conditions within the magmatic system e.g.^29^, but this is unlikely to have controlled all the observed changes.

### Genetic implications for porphyry deposit-forming magmatic systems

The apparent change in geochemistry (whole-rock and zircon; Figs. 5, 6, 7, 8) as the Yerington system began to produce porphyry deposits is consistent with observations from a wide range of similar magmatic centres globally where precursor magmatism and syn-mineralisation intrusions have been examined e.g.^19,22,24,26,29,84^. Typically, these changes have been interpreted solely as being due to long-term, tectonically driven arc-scale, transitional processes over millions of years or ‘ramp-ups’ towards ore-formation e.g.^11,16,21,23^. However, these explanations are relatively poorly constrained due to limited exposure in most porphyry systems^30^. From our studies of the well exposed, ~ 8 km deep cross section through the Yerington system, the possibility exists for a much more rapid (< 200 kyrs) shift to porphyry deposit-forming magmatism. Significant changes in geochemical signatures over relatively short timescales at the transition to porphyry deposit forming magmatism have been indicated elsewhere e.g.^24,84^, but this study provides unprecedented temporal and spatial resolution due to the acquisition of our new high precision geochronological framework and the unique depth constraints at Yerington. This short timeframe does not necessarily contradict the suggestion of longer-term progressions towards ore-forming arc magmas, commonly seen in other systems. Rather it captures how rapid changes in the ore-forming potential of the magmatic system may occur. In other porphyry centres, the rapid timescales are often inconspicuous due to the limited rock record available. As such, the much longer durations between precursor and ore-related magmatism documented elsewhere, alongside their corresponding change in geochemistry, may relate to differences in the juxtaposition of upper crustal magmatic expressions over the protracted duration of the magmatic system. For example, when only the shallow levels (e.g. ~ 2 km palaeodepth) are considered at Yerington, porphyry dykes that share a comparable magmatic evolution to the LHG could yield an apparent temporal difference of ~ 1.7 Myrs with the Bear QM they cross-cut. The timescale of the change in geochemical signatures between these units will appear drawn-out and to have developed over longer timescales, whereas at depth the system is demonstrably more concurrent.

The recognition of rapid changes within the magmatic plumbing system requires a new perspective when interpreting magmatic processes in ore-forming systems. Because the magmas responsible for ore-formation underwent different routes of evolution and likely were tapped from spatially independent, deeper melt zones, it suggests the processes and evolution histories of early-intruded plutons cannot necessarily be used to infer whether other parts of the batholith may have produced porphyry-type deposits, and we advise caution over the use of earlier parts of the magmatic system to infer the nature of what has been removed or added to the melts over longer time periods or apparent progressions of melt chemistry such as metal contents that may be removed by earlier sulphide fractionation e.g.^85^. With lower precision geochronology (e.g. 2% typical of microbeam U–Pb methods), these earlier intrusive phases that emanate from potentially disparate magmatic plumbing systems may even appear to be ‘coeval’ with mineralisation.

The short, < 200 kyrs timescale for the emergence of the geochemical signatures associated with mineralisation that appeared throughout the magmatic system (in plutons, dykes and volcanics) significantly narrows and better defines the temporal footprint that can be used to identify ore-forming processes within the rock record. This has significance in the development and refinement of porphyry exploration indicators by increasing the potential spatio-temporal efficacy of using these geochemical ‘fertility’ signatures to isolate areas most prospective for porphyry-style mineralisation. Whilst the large-scale long-duration, tectonically driven signatures previously identified can still be critical in defining general targets, increased resolution by which the ore forming signature can be discriminated can lead to greater confidence in identifying and discovering the next generation of porphyry deposits, which are likely to be deeper and often under cover and so will be more difficult to find^10^.

## Conclusions

From this geochemical and geochronological study of the unique and archetypal Yerington magmatic system, the timing of batholith construction, magmatic evolution and magmatic-hydrothermal porphyry-style mineralisation has been constrained. These findings provide an improved framework for new conceptual models for magmatic systems and batholith construction, porphyry mineralisation and the relationships with volcanism. By doing so we have identified a rapid shift in whole-rock and zircon geochemistry at the onset of porphyry mineralisation. This is attributed to a change from extraction of magmas from mid-crustal reservoirs, to form the McLeod QMD and Bear QM, that had relatively poor ore-forming potential, to extraction of volatile-rich granitic melts from greater (~ 30 km) depths which were emplaced at shallow levels to form the LHG (~ 3–8 km). As the LHG crystallised, late stage melts were emplaced through the carapace as aplite dykes; these provided conduits for the transport of mineralising fluids into the zone of ore formation. The rapidity (< 200 kyrs) of the change in the magmatic system shows that geochemical signatures of certain plutonic and volcanic rocks can be used to interpret the specific magmatic process that eventually lead to porphyry mineralisation. Isolating short-timescale changes in magmatic plumbing, and melt chemistry, in addition to the longer, tectonically-driven multi-million-year timescales of arc-scale magmatic evolution, allows for the refinement and increased efficacy of geochemical ‘fertility indicator’ tools that extend into the volcanic environment, and aid exploration for the next generation of ore deposits.

## Methods

The fundamental first-order controls for this study are from field observations of cross-cutting relationships, disequilibrium phenomena and textures indicative or fluid exsolution and mineralisation in outcrop across the Yerington magmatic system and drill core from the Ann Mason and Yerington porphyry copper deposits. Based on these observations, select samples (full list in Supplementary Data 1) were chosen for petrographic study (including QEMSCAN, Supplementary Data 3), whole-rock XRF and ICP-MS analysis (Supplementary Data 4), zircon LA-ICP-MS (Supplementary Data 5) analysis, zircon U–Pb CA–ID–TIMS and Re-Os molybdenite geochronology (Supplementary Data 2) and zircon Lu–Hf isotope analysis (Supplementary Data 6).

### QEMSCAN

Select samples for the petrographic study were prepared as polished uncovered thin sections (30 μm thickness) at Camborne School of Mines, University of Exeter, UK. Following optical microscopy, the mineralogy of select thin sections was determined using a QEMSCAN 4300 automated mineralogical assessment system at Camborne School Mines, using the same methods that are described in detail in^33^. The data were output as mineralogical maps (Fig. S4–S6) with accompanying numerical data (Supplementary Data 3).

### Electric pulse fragmentation (EPF)

Initial sample preparation for subsequent whole-rock and zircon analyses was by electric pulse fragmentation (EPF), also known as electrodynamic fragmentation (EDF), electrical disintegration (ED) and electric pulse disaggregation (EPD)^86,87^. EPF utilises highly energetic (150–750 J/pulse) pulsed electrical discharges with a fast pulse rise time (< 500 ns) to break composite materials apart along internal compositional or mechanical boundaries. Samples are submerged in a dielectric process medium such as water, which is more resistive than solids at these pulse rise times, resulting in the discharge being forced through the relatively conductive solid and along internal phase boundaries such as mineral–mineral contacts. Each discharge event is a movement of electrons from the working electrode to the ground electrode as a plasma channel^86,88^. The rapid formation of this plasma channel causes explosive expansion within the material along the discharge pathway^86,87^. In addition to direct breakage from the plasma channel, this explosion creates a shockwave that propagates through the material. Varying elasticity moduli between minerals results in shear stresses being focussed on mineral contact surfaces, causing intra-mineral breakage and disaggregating the rock. This tensile intra-mineral breakage is less damaging to individual minerals that are liberated from the rock larger and more intact than mechanical crushing.

The treatment was conducted using the ‘Lab’, a laboratory scale EPF device for the batch processing of material, manufactured by SELFRAG AG, Switzerland. The Lab is designed to process samples of up to approximately 1 L volume, or single particles with a top passing size of 40–45 mm in a 4 L process vessel filled with de-mineralized water. It produces high voltage (90–200 kV) electric discharges of short duration between two electrodes: the ‘working’ electrode is immersed in the upper part of the process vessel, while the bottom of the vessel constitutes the ‘counter/grounding’ electrode. The operating parameters that can be changed are the discharge voltage, electrode gap, pulse repetition rate and number of electric pulses applied to the sample, with treatment conditions for this work listed in Table 1. Further information on the Lab system can be found in^89^.

**Table 1:** Treatment conditions for electric pulse fragmentation.

Vessel (open/closed)	Sieve aperture	Voltage	Pulse repetition rate	Electrode gap	Pulses per cycle
Open	2 mm	180 kV	5 Hz	40 mm	100

Samples were manually crushed to 40–45 mm to fit into the process vessel. From optical studies the zircons have an average grain diameter of ~ 200 microns, which guided selection of an appropriate aperture sieve for the SELFRAG open process vessel. Appropriate sieve aperture diameter is generally equal to 10 × the target particle diameter. A series of 100 pulses were applied to the sample followed by visual inspection of the remaining sample; if > 10% if the sample remained above the sieve, another cycle of 100 pulses were administered. When > 90% of sample material had passed through the sieve, treatment was stopped, and the sample recovered from the process vessel collection cup before drying at 70 °C.

### Whole-rock XRF and ICP-MS analysis, and CIPW normative mineralogy

Fully quantitative X-ray fluorescence (XRF) for whole-rock geochemistry was performed at the University of Leicester’s Department of Geology on a PANalytical Axios Advanced XRF spectrometer. Major elements were determined on fused glass beads (prepared from ignited powders; sample to flux ratio 1:5, 80% Li metaborate: 20% Li tetraborate flux) and trace elements were analysed on pressed powder briquettes (32 mm diameter; 7.5 g sample mixed with 15–20 drops 7% PVA solution as binding agent, pressed at 10 tons per sq. inch). Major element results were quoted as component oxide weight percent, re-calculated to include loss on ignition (LOI). Information on the standards analysed and the accuracy and precision of the XRF analysis is available in Supplementary Data 4.

Inductively coupled plasma mass spectrometry (ICP-MS) for whole-rock trace element geochemistry was also performed at the University of Leicester’s Department of Geology on a ThermoScientific ICAP-Qc quadrupole ICP mass spectrometer. Analysis for rare earth elements (REEs), Hf, Sr and Y was performed on solution from the same fused glass beads used for XRF analysis. Information on the standards analysed and the accuracy and precision of the ICP-MS analysis is available in Supplementary Data 4.

Whole-rock XRF geochemistry was used to calculate CIPW normative mineralogy (method of^90^, after^91^). Normative mineralogy data was then plotted on the H_2_O-saturated melt minima ternary plot of^78^ (after^77^) to estimate the pressures of melt differentiation^67^. Assuming lithostatic conditions, pressures from this plot were used to equate approximate depths of melt differentiation using *P* = *ρgh* and assuming an average overburden density of 2.5 g/cm^3^. We suggest that these depths only be used as approximations. Samples with very little apparent orthoclase either do not represent H_2_O saturated melts, have complex crystal cargoes, or were subject to overprinting by Na–Ca and/or propylitic hydrothermal alteration (as previously mapped e.g.^47,92^), and were not used in our depth approximations.

### Zircon separation

Zircons were separated from disaggregated samples at the British Geological Survey, Keyworth, using the sequentially described circuit: Sieve to < 500 μm using a Fritsch automatic sieve; Pass the < 500 μm fraction over a Gemini water table, twice; Separate non-magnetic minerals using a Frantz isodynamic separator—subsequent paramagnetic charges of 0.1 A, 0.3 A, 0.7 A, 1.1 A and 1.7 A were used to reduce the bulk material in stages; Perform gravity separation utilising methylene iodide (ca. 3.32 SG) as a density medium. The final zircon (amongst other phases) separate was thermally annealed at 900 °C for 12 h. Annealed zircon grains were then picked by hand and prepared as polished blocks. Cathodoluminescence (CL) images of these were generated by SEM-CL, using an FEI Quanta 650F FEG-SEM equipped with a Gatan monochrome CL detector at the University of Exeter’s Environment and Sustainability institute operating at an accelerating voltage of 20 kV, as well as using a CITL Mk5 electron source, operating at approximately 250 μA and 10 kV. For the latter, images were captured using a Nikon DS-Ri2 camera, attached to a petrographic microscope, and operated using NiS-elements software. Images were captured in a darkened room, with an exposure time of 2 s.

### Zircon LA–ICP–MS

Zircon cores and rims were analysed for their trace element geochemistry in the LODE Laboratory at the Natural History Museum, London, using an ESI (New Wave Research) NWR193 excimer laser coupled to an Agilent 7700 × quadrupole ICP-MS. Individual zircon grains were located using images obtained by cold-cathode CL and SEM-CL at Camborne School of Mines. A spot size of 30 μm was used and ablation was performed at a repletion rate of 5 Hz and fluence of 3.5 J/cm^2^. For each spot, approximately 20 s of background signal followed by 40 s of signal acquisition during ablation. Analytical conditions, including isotopes measured and dwell times are summarised in Supplementary Data 5.

### Zircon U–Pb CA–ID–TIMS geochronology

Chemical abrasion isotope dilution thermal ionization mass spectrometry (CA–ID–TIMS) U–Pb zircon geochronology was undertaken at the Geochronology and Tracers Facility, British Geological Survey, Keyworth. After thermal annealing at 900 °C zircon were chemically abraded at 190 °C for 12 h following^93^. The methodology for all other analytical procedures, instrumental conditions, corrections and data reduction follows that outlined in detail in^72^ using the ET(2)535 tracers^94,95^. Isotope ratio measurements were made using a Thermo Triton thermal ionization mass-spectrometer (TIMS), with the U decay constants of^96^, the ^238^U/^235^U ratio of^97^, and the decay constants for ^230^Th of^98^. The ^206^Pb/^238^U dates were corrected for initial ^230^Th disequilibrium^99^ upon zircon crystallisation using the zircon/melt partition coefficient *f*_Th/U_ of 0.246^100^. Results are reported in Supplementary Data 2.

The estimates of igneous crystallisation ages after emplacement are selected from the ^206^Pb/^238^U (Th corrected) weighted mean of the youngest population of data where the date had a statistically acceptable MSWD (Mean square of weighted deviates) for the given population size and attributed 2σ uncertainties, indicating that any dispersion between the selected analyses can be attributable to the measurement of a single population. All samples, with the exception of the volcanic sample BS1, display over-dispersion between the dates of individual zircons or zircon fragment dates in excess of that expected due to analytical scatter. Dates that are older than the statistically valid weighted mean single population are attributed to antecrystic zircon growth, either being sourced from deeper within the magmatic system than the emplacement level or due to protracted crystallisation of zircon upon emplacement. To further evaluate the sensitivity of the age interpretation to the selection of dates we evaluated two further scenarios of date calculation: 1) Selecting the youngest date as being representative of youngest zircon growth; 2) selecting the weighted mean date of the youngest three dates that give a statistically acceptable MSWD. These evaluations of date selections are provided in Supplementary Data 2, and illustrate that regardless of the approach adopted the timescales we discuss are robust.

When comparing dates either internally or to other data sets that are undertaken with the Earthtime mixed U–Pb tracers^94,95^ only the analytical uncertainties need to be considered. To evaluate U–Pb dates against other isotopic systems, systematic uncertainties must also be acknowledged within the interpretation. The total uncertainty including systematic components from tracer calibration and decay constants are provided with age interpretations in Supplementary Data 2. For comparison with the Re-Os dates where they include the Re-Os decay constant uncertainty we recommend that only the tracer calibration uncertainty is considered for the U–Pb data as λ^187^Re is derived from inter-calibration with U–Pb data^101,102^.

### Zircon Lu–Hf isotopes

The Lu–Hf fractions were obtained from elements eluted under 3 M HCl within the ion exchange U and Pb purification scheme during CA–ID–TIMS U–Pb analysis e.g.^103^. Results of the Lu–Hf isotope analysis (Supplementary Data 6) therefore correspond to the same volume of material as the associated zircon U–Pb date. Selecting zircon from the young weighted mean population provides temporal constraints that the volume best captures the nature of the melt upon emplacement. The Lu and Hf elution was dried at 70 °C to a chloride before being dissolved in 1 ml of 2% HNO_3_ + 0.1 M HF, prior to analysis on a Thermo-Electron Neptune Plus mass spectrometer, using a Cetac Aridus II desolvating nebuliser. 0.006 l/min of nitrogen were introduced via the nebulizer in addition to Ar in order to minimise oxide formation. The instrument was operated in static multicollection mode, with cups set to monitor ^172^Yb, ^173^Yb, ^175^Lu, ^176^Lu + Hf + Yb, ^177^Hf, ^178^Hf, ^179^Hf and ^180^Hf. 1% dilutions of each sample were tested prior to analysis, and samples diluted to ca. 20 ppb. Standard sample cones and X-skimmer cones were used, giving a typical signal of ca. 800–1000 V/ppm Hf. Correction for ^176^Yb on the ^176^Hf peak was made using reverse-mass-bias correction of the ^176^Yb/^173^Yb ratio empirically derived using Hf mass bias corrected Yb-doped JMC475 solutions^104^. ^176^Lu interference on the ^176^Hf peak was corrected by using the measured ^175^Lu and assuming ^176^Lu/^175^Lu = 0.02653. Data are reported relative to ^179^Hf/^177^Hf = 0.7325. The Hf standard solution JMC475 was analysed during each analytical session and sample ^176^Hf/^177^Hf ratios are reported relative to a value of 0.282160 for this standard^104^. Eleven analyses of JMC475 gave a mean ^176^Hf/^177^Hf value of 0.282146 ± 0.000007 (1σ). Typical external precision was in the range between 13 and 22 ppm. Data were reduced with an in-house calculation and time corrected values include uncertainty propagated from the weighted mean U–Pb date of the sample.

### Rhenium–osmium molybdenite geochronology

Molybdenite Re-Os ages were determined for quartz-chalcopyrite-bornite-molybdenite-quartz veins (samples AC11, AC12 & AC21), a chalcopyrite-molybdenite bearing quartz UST (or vein-dyke texture e.g.^53–55^) within an aplite dyke (sample AC3) and a fine grained molybdenite vein (sample AC41MP) sampled from drill core from the Ann Mason porphyry deposit. Sample details in Supplementary Data 2 and Fig. S8.

The Re-Os molybdenite analysis were carried out in the Source Rock and Sulfide Geochemistry and Geochronology, and Arthur Holmes Laboratories at University of Durham (United Kingdom) to establish the Re-Os age of molybdenite mineralisation. A total of eight analyses were conducted. One from samples AC3, AC11, AC12 & AC21 and four from sample AC41MP (For which sample AC41MP was approximately split into four equal subsamples. This offers the opportunity to check the consistency and closed behaviour of the Re-Os system within the vein). Pure molybdenite separates were obtained from the silicate matrix was achieved using the HF purification method^105^, and then further purified (removal of any pyrite, chalcopyrite and/or bornite and undissolved silicate phases) by hand under a binocular microscope.

An aliquant of the molybdenite separate (~ 20 mg) together with a known amount of tracer solution (^185^Re + Os bearing a normal isotope composition) were placed into a carius tube and digested with 3 mL HCl and 6 mL HNO_3_ at 220 °C for 24 h. Osmium was isolated and purified using solvent extraction (CHCl_3_) and micro-distillation methods, with the resulting Re-bearing fraction purified using NaOH-Acetone solvent extraction and anion chromatography^83,106^. Although negligible in comparison to the Re and Os abundance in the molybdenite, the final Re-Os data are blank corrected. A full analytical protocol blank run parallel with the molybdenite analysis yields 3.9 pg Re and 0.5 pg Os, the latter possessing a ^187^Os/^188^Os composition of 0.21 ± 0.2. Data treatment follows that outlined in^83^. Osmium mass fractionation was monitored in real time by repeatedly determining the Os isotope composition of the tracer and correcting this to a value of 3.08761 for ^192^Os/^188^Os. The isotopic composition of Re was corrected for instrumental fractionation from the difference between the Re data obtained for the standard and the value of ^185^Re/^187^Re = 0.59738^107^. The data are also oxide corrected, as carried out in many other studies e.g.^83,108^. All Re-Os data are given with 2σ absolute uncertainties (Supplementary Data 2). Molybdenite Re-Os ages are calculated using a ^187^Re decay constant of 1.666 × 10^−11^ *y*^*-1*^ with an uncertainty of 0.31%^101,102^. The Henderson molybdenite reference material (RM8599) analyzed during the course of this study yields a Re-Os age of 27.62 ± 0.11 (2σ; *n* = 1), which is in good agreement with the recommended value of 27.66 ± 0.10 Ma^108,109^, and that reported by^83^ (27.695 ± 0.038 Ma, *n* = 9) and previous analysis at Durham (e.g., 27.65 ± 0.12 Ma;^105^ and references therein).

## Supplementary Information

Supplementary Information 1.

Supplementary Information 2.

Supplementary Information 3.

Supplementary Information 4.

Supplementary Information 5.

Supplementary Information 6.

Supplementary Information 7.

## Data Availability

All data supporting the findings of this study are available within the paper and its Supplementary files.

## References

[1] Jowitt SM, Mudd GM, Thompson JFH (2020). Future availability of non-renewable metal resources and the influence of environmental, social, and governance conflicts on metal production. Commun. Earth Environ..

[2] Herrington R (2021). Mining our green future. Nat. Rev. Mater..

[3] Elshkaki A, Graedel TE, Ciacci L, Reck BK (2016). Copper demand, supply, and associated energy use to 2050. Glob. Environ. Chang..

[4] Watari T, Nansai K, Nakajima K (2021). Major metals demand, supply, and environmental impacts to 2100: A critical review. Resour. Conserv. Recycl..

[5] Sillitoe RH (2010). Porphyry Copper Systems. Econ. Geol..

[6] Richards, J. P. Cumulative factors in the generation of giant calc-alkaline porphyry Cu deposits. *In:* Porter, T.M. (ed.) Super porphyry copper and gold deposits: A global perspective. v. 1: Adelaide, PGC Publishing 7−25 (2005).

[7] Wilkinson JJ (2013). Triggers for the formation of porphyry deposits in magmatic arcs. Nat. Geosci..

[8] Richards JP (2015). Tectonic, magmatic, and metallogenic evolution of the Tethyan orogen: From subduction to collision. Ore Geol. Rev..

[9] Chiaradia M, Caricchi L (2017). Stochastic modelling of deep magmatic controls on porphyry copper deposit endowment. Sci. Rep..

[10] Cooke, D. R., Agnew, P., Hollings, P. *et al.* Porphyry indicator minerals (PIMS) and porphyry vectoring and fertility tools (PVFTS) – Indicators of mineralisation styles and recorders of hypogene geochemical dispersion halos. In: Tschirhart, V. & Thomas, M. D. (eds.) Proceedings of Exploration 17: Sixth Decennial International Conference on Mineral Exploration 457–470 (2017).

[11] Rohrlach B, Loucks R (2005). Multi-million-year cyclic ramp-up of volatiles in a lower crustal magma reservoir trapped below the Tampakan copper-gold deposit by MIO-PLIOCENE crustal compression in the southern Philippines. Adelaide, PGC Publ..

[12] Richards JP (2011). High Sr/Y arc magmas and porphyry Cu±Mo±Au deposits: Just add water. Econ. Geol..

[13] Chiaradia M, Ulianov A, Kouzmanov K (2012). Why large porphyry Cu deposits like high Sr/Y magmas?. Sci. Rep..

[14] Chiaradia M (2015). Crustal thickness control on Sr/Y signatures of recent arc magmas: an Earth scale perspective. Sci. Rep..

[15] Richards JP (2012). High Sr/Y magmas reflect Arc maturity, high magmatic water content, and porphyry Cu ± Mo ± Au potential: examples from the Tethyan arcs of central and Eastern Iran and Western Pakistan. Econ. Geol..

[16] Loucks RR (2014). Distinctive composition of copper-ore-forming arc magmas. Aust. J. Earth Sci..

[17] Lu Y, Loucks RR, Fiorentini M (2016). Zircon compositions as a pathfinder for porphyry Cu ± Mo ± Au deposits. Soc. Econ. Geologi. Spec Publ. Series.

[18] Williamson B, Herrington R, Morris A (2016). Porphyry copper enrichment linked to excess aluminium in plagioclase. Nature Geosci..

[19] Nathwani CL, Loader MA, Wilkinson JJ (2020). Multi-stage arc magma evolution recorded by apatite in volcanic rocks. Geology.

[20] Lee RG, Byrne K, D’Angela M (2021). Using zircon trace element composition to assess porphyry copper potential of the Guichon Creek batholith and Highland Valley Copper deposit, south-central British Columbia. Miner. Deposita.

[21] Ballard JR, Palin JM, Campbell IH (2002). Relative oxidation states of magmas inferred from Ce(IV)/Ce(III) in zircon: application to porphyry copper deposits of northern Chile. Contrib. Mineral. Petrol..

[22] Chiaradia M, Meino D, Spikings R (2009). Rapid transition to long-lived deep crustal magmatic maturation and the formation of giant porphyry-related mineralization (Yanacocha, Peru). Earth. Planet. Sci. Lett..

[23] Rezeau H, Moritz R, Wotzlaw J (2016). Temporal and genetic link between incremental pluton assembly and pulsed porphyry Cu-Mo formation in accretionary orogens. Geology.

[24] Nathwani CL, Simmons AT, Large SJE (2021). From long-lived batholith construction to giant porphyry copper deposit formation: Petrological and zircon chemical evolution of the Quellaveco District. Southern Peru. Contrib. Mineral. Petrol..

[25] Richards JP (2003). Tectono-magmatic precursors for porphyry Cu-(Mo-Au) deposit formation. Econ. Geol..

[26] Lee CTA, Tang M (2020). How to make porphyry copper deposits. Earth Planet. Sci. Lett..

[27] Dilles JH (1987). Petrology of the Yerington Batholith, Nevada: Evidence for evolution of porphyry copper ore fluids. Econ. Geol..

[28] Seedorff E (2005). Porphyry deposits: Characteristics and origin of hypogene features. Econ. Geol..

[29] Dilles JH, Kent AJR, Wooden JL (2015). Zircon compositional evidence for sulfur-degassing from ore-forming arc magmas. Econ. Geol..

[30] Seedorff E, Barton MD, Stavast WJA, Maher DJ (2008). Root zones of porphyry systems: Extending the porphyry model to depth. Econ. Geol..

[31] Weis P, Driesner T, Heinrich CA (2012). Porphyry-copper ore shells form at stable pressure-temperature fronts within dynamic fluid plumes. Science.

[32] Schöpa A, Annen C, Dilles JH, Sparks RSJ, Blundy JD (2017). Magma emplacement rates and porphyry copper deposits: Thermal modeling of the Yerington Batholith Nevada. Econ. Geol..

[33] Carter LC, Williamson BJ, Tapster SR (2021). Crystal mush dykes as conduits for mineralising fluids in the Yerington porphyry copper district. Nevada. Commun. Earth Environ..

[34] Proffett, J. M. & Dilles, J. H. Geological map of the Yerington district, Nevada: Nevada Bureau of Mines and Geology, map 77 (1984).

[35] Dilles JH, Wright JE (1988). The chronology of early Mesozoic arc magmatism in the Yerington district of western Nevada and its regional implications. GSA Bull..

[36] Banik TJ, Coble MA, Miller CF (2017). Porphyry Cu formation in the middle Jurassic Yerington batholith, Nevada, USA: Constraints from laser Raman, trace element, U-Pb age, and oxygen isotope analyses of zircon. Geosphere.

[37] Speed, R. C. Paleogeographic and plate tectonic evolution of the early Mesozoic marine province of the western Great Basin. *In* Howell D. G., and McDougall, K. A. (eds.), Mesozoic paleogeography of the western United States, Pacific Coast Paleogeography Symposium 2: Soc. Econ Paleontologist Mineralogists, Pacific Sec., 1978, Sacramento, California 253–270(1978).

[38] Proffett, J. M. Report on the geology and genesis of the Yerington porphyry copper district, Nevada, a four dimensional study. *Final report for: USGS mineral resource external research program grant 06HQGR0171* (2007).

[39] Proffett JM (1977). Cenozoic geology of the Yerington district, Nevada, and implications for the nature and origin of Basin and Range faulting. GSA Bull..

[40] Proffett JM (2009). High Cu grades in porphyry Cu deposits and their relationship to emplacement depth of magmatic sources. Geology.

[41] Proffett, J. M. Ore deposits of the western United States: A summary. *In:* Ridge, J. D. (ed.) Papers on Mineral Deposits of Western North America: Nevada Bureau of Mines and Geology Report 33 13–32 (1979).

[42] Dilles, J. H. & Proffett, J. M. Metallogenesis of the Yerington batholith, Nevada. *In:* Pierce, F. W., & Bolm, J. G. (Eds.), Porphyry copper deposits of the American cordillera: Arizona Geological Society Digest, **20** 306–315 (1995).

[43] Bryan, R. C. NI 43-101 Technical Report Mineral Resource Update Yerington Copper Project Lyon County, Nevada. *TetraTech* (2014). Available: https://www.sec.gov/Archives/edgar/data/1339688/000106299314000036/exhibit99-1.htm Accessed 22/11/2021.

[44] Hudbay Minerals Inc. Mason Preliminary Economic Assessment Summary (2021). Available: https://hudbayminerals.com/investors/press-releases/press-release-details/2021/Hudbay-Announces-Positive-Preliminary-Economic-Assessment-for-its-Mason-Copper-project/default.aspx Accessed 04/10/2021.

[45] Welhener, H., Woods, J. & Dischler, S. MacArthur Copper Project, Mason Valley, Nevada, USA. NI 43–101 Technical Report. Mineral Resource Estimate. *Independent Mining Consultants, Inc.* (2022). Available: https://www.lioncg.com/wp-content/uploads/2022/02/MacArthur_mineral-resource_2022-02-25.pdf Accessed 21/09/2022.

[46] Carten RB (1986). Sodium-calcium metasomatism: Chemical, temporal, and spatial relationships at the Yerington, Nevada. Porphyry Copper Deposit. Econ. Geol..

[47] Dilles JH, Einaudi MT, Proffett J, Barton MD (2000). Overview of the yerington porphyry copper district: Magmatic to nonmagmatic sources of hydrothermal fluids, their flow paths, alteration affects on rocks, and Cu-Mo-Fe-Au ores. Soc. Econ Geologi. Guideb. Ser..

[48] Runyon SE, Steel-MacInnis M, Seedorff E (2017). Coarse muscovite veins and alteration deep in the Yerington batholith, Nevada: Insights into fluid exsolution in the roots of porphyry copper systems. Miner. Deposita.

[49] Runyon SE (2019). Coarse muscovite veins and alteration in porphyry systems. Ore Geol. Rev..

[50] Candela PA (1997). A review of shallow, ore-related granites: Textures, volatiles, and ore metals. J. Petrol..

[51] Gustafson LB, Hunt JP (1975). The porphyry copper deposit at El Salvador Chile. Econ. Geol..

[52] Carter LC, Williamson BJ (2022). Textural indicators of mineralisation potential in porphyry magmatic systems – A framework from the archetypal Yerington district Nevada. Ore Geol. Rev..

[53] Spurr, J. E. (1923) The ore magmas: McGraw-Hill. New York 915

[54] Shannon JR, Walker BM, Carten RB, Geraghty EP (1982). Unidirectional solidification textures and their significance in determining relative ages of intrusions at the Henderson Mine Colorado. Geology.

[55] Kirkham, R. V. & Sinclair, W. D. Comb quartz layers in felsic intrusions and their relationship to porphyry deposits. *In:* Taylor, R. P. & Strong, D. F. (eds.), Recent advances in the geology of granite-related mineral deposits. Canadian Institute of Mining, Special Volume 39, 50–71 (1988).

[56] London D (2009). The origin of primary textures in granitic pegmatites. Can. Mineral..

[57] Kirwin, D. J. Unidirectional solidification textures associated with intrusion-related Mongolian mineral deposits. *In:* Seltmann, R., Gerel, O., & Kirwin, D.J., (eds.), Geodynamics and metallogeny of Mongolia with special emphasis on copper and gold deposits: Society of Economic Geologists-International Association for the Genesis of Ore Deposits Field Trip, 2005: IAGOD Guidebook Series 11: London, Centre for Russian and Central EurAsian Mineral Studies, Natural History Museum, 63−84 (2005).

[58] Candela, P. A. Felsic magmas, volatiles, and metallogenesis. *In:* Whitney, J. A. & Naldrett, A. J. (Eds.), Ore Deposition Associated with Magmas. *Reviews in Economic Geology*, 223–233 (1989).

[59] Menand T (2011). Physical controls and depth of emplacement of igneous bodies: A review. Tectonophysics.

[60] Luhr JF, Carmicheal ISE, Varekamp JC (1984). The 1982 eruptions of El Chichón Volcano, Chiapas, Mexico: Mineralogy and petrology of the anhydritebearing pumices. J. Volcanol. Geotherm..

[61] Nandedkar RH, Hürlimann N, Ulmer P, Müntener O (2016). Amphibole–melt trace element partitioning of fractionating calc-alkaline magmas in the lower crust: an experimental study. Contrib. Mineral. Petrol..

[62] Aigner-Torres M, Blundy J, Ulmer P, Pettke T (2007). Laser Ablation ICPMS study of trace element partitioning between plagioclase and basaltic melts: An experimental approach. Contrib. Mineral. Petrol..

[63] Sisson TW (1994). Hornblende-melt trace-element partitioning measured by ion microprobe. Chem. Geol..

[64] Davidson J, Turner S, Handley H (2007). Amphibole sponge in arc crust?. Geology.

[65] Claiborne LL, Miller CF, Walker BA (2006). (2006) Tracking magmatic processes through Zr/Hf rations in rocks and Hf and Ti zoning in zircons: An example from the Spirit Mountain batholith. Nevada. Mineral. Mag..

[66] Burnham A (2020). Key concepts in interpreting the concentrations of the rare earth elements in zircon. Chem. Geol..

[67] Annen C, Blundy JD, Sparks RSJ (2006). The genesis of intermediate and silicic magmas in deep crustal hot zones. Jour. Petrol..

[68] Burnham AD, Berry AJ (2012). An experimental study of trace element partitioning between zircon and melt as a function of oxygen fugacity. Geochim. Cosmochim. Acta.

[69] Loucks RR, Fiorentini ML, Henríquez GJ (2020). New magmatic oxybarometer using trace elements in zircon. J.Petrol..

[70] Loader MA, Wilkinson JJ, Armstrong RN (2017). The effect of titanite crystallisation on Eu and Ce anomalies in zircon and its implications for the assessment of porphyry Cu deposit fertility. Earth Planet. Sci. Lett..

[71] Watson EB, Harrison TM (2005). Zircon thermometer reveals minimum melting conditions on earliest earth. Science.

[72] Tapster S, Condon DJ, Naden J (2016). Rapid thermal rejuvenation of high-crystallinity magma linked to porphyry copper deposit formation; evidence from the Koloula Porphyry Prospect. Solomon Islands. Earth. Planet. Sci. Lett..

[73] Taylor HP (1980). The effects of assimilation of country rocks by magmas on 18O/16O and 87Sr/86Sr systematics of igneous rocks. Earth. Planet. Sci. Lett..

[74] Valley JW (2003). Oxygen isotopes in zircon. Rev. Mineral. Geochem..

[75] Bindeman I (2008). Oxygen isotopes in mantle and crustal magmas as revealed by single crystal analysis. Rev. Mineral. Geochem..

[76] Smith D (2014). Clinopyroxene precursors to amphibole sponge in arc crust. Nat. Commun..

[77] Johannes, W. & Holtz, F. The Haplogranite System Qz-Ab-Or. *In:* Petrogenesis and Experimental Petrology of Granitic Rocks. Minerals and Rocks, vol 22. Springer, Berlin, Heidelberg (1996). 10.1007/978-3-642-61049-3_2

[78] Blundy J, Cashman K (2001). Ascent-driven crystallisation of dacite magmas at Mount St Helens, 1980–1986. Contrib. Mineral. Petrol..

[79] Romero BK, S, Wong, C. (2011). Molybdenite mineralization and Re-Os geochronology of the Escondida and Escondida Norte porphyry deposits. Northern Chile. Resour. Geol..

[80] Stein HJ, Holland HD, Turekian KK (2014). Dating and tracing the history of ore formation. Treatise on geochemistry, second edition 13.

[81] Spencer ET, Wilkinson JJ, Creaser RA, Seguel J (2015). The distribution and timing of molybdenite mineralization at the El Teniente Cu-Mo porphyry deposit Chile. Econ. Geol..

[82] Chang J, Li J-W, Selby D, Liu J-C, Deng X-D (2017). Geological and chronological constraints on the long-lived Eocene Yulong porphyry Cu-Mo deposit, eastern Tibet, China: Implications for lifespan of magmatic-hydrothermal processes forming giant and supergiant porphyry Cu deposits. Econ. Geol..

[83] Li Y, Selby D, Condon D, Tapster S (2017). Cyclic magmatic-hydrothermal evolution in porphyry systems: High-precision U-Pb and Re-Os geochronology constraints from the Tibetan Qulong porphyry Cu-Mo deposit. Econ. Geol..

[84] D’Angelo M, Alfaro M, Hollings P (2017). Petrogenesis and magmatic evolution of the Guichon creek batholith: Highland valley porphyry Cu ± (Mo) district, south-central British Columbia. Econ. Geol..

[85] Park JW, Campbell IH, Malaviarachchi SPK (2019). Chalcophile element fertility and the formation of porphyry Cu ± Au deposits. Miner. Deposita.

[86] Andres U, Jirestig J, Timoshkin I (1999). Liberation of minerals by high-voltage electrical pulses. Powder Technol..

[87] Bluhm H, Frey W, Giese H (2000). Application of pulses HV discharges to material fragmentation and recycling. IEEE Trans. Dieletr. Electr. Insul..

[88] van der Wielen, K. P. Application of high voltage breakage to a range of rock types of varying physical properties. Ph.D. thesis, Camborne School of Mines, University of Exeter, UK (2013).

[89] Bru K, Beaulieu M, Sousa R (2020). Comparative laboratory study of conventional and Electric Pulse Fragmentation (EPF) technologies on the performances of the comminution and concentration steps for the beneficiation of a scheelite skarn ore. Miner. Eng..

[90] Lowenstern, J. B. C.I.P.W. norm. Calculator. *USGS* (2000). https://volcanoes.usgs.gov/observatories/yvo/jlowenstern/other/software_jbl.html

[91] Kelsey CH (1965). Calculation of the C.I.P.W. norm. Mineral. Mag..

[92] Halley S, Dilles JH, Tosdal JH (2015). Footprints: Hydrothermal alteration and geochemical dispersion around porphyry copper deposits. SEG Newsl..

[93] Mattinson JM (2005). Zircon U-Pb chemical abrasion (“CA-TIMS”) method: Combining annealing and multi-step partial dissolution analysis for improved precision and accuracy of zircon ages. Chem. Geol..

[94] Condon DJ, Schoene B, McLean NM, Bowring SA, Parrish RR (2015). Metrology and traceability of U-Pb isotope dilution geochronology (EARTHTIME tracer calibration part I). Geochim. Cosmochim. Acta..

[95] McLean NM, Condon DJ, Schoene B, Bowring SA (2015). Evaluating uncertainties in the calibration of isotopic reference materials and multi-element isotopic tracers (EARTHTIME tracer calibration part II). Geochim. Cosmochim. Acta..

[96] Jaffey AH, Flynn KF, Glendenin LE (1971). Precision measurement of half-lives and specific activities of U-235 and U-238. Phys. Rev..

[97] Hiess J, Condon DJ, McLean N, Noble SR (2012). U-238/U-235 Systematics in terrestrial uranium-bearing minerals. Science.

[98] Cheng H, Edwards RL, Hoff J (2000). The half-lives of uranium-234 and thorium-230. Chem. Geol..

[99] Schärer U (1984). The effect of initial 230Th disequilibrium on young U-Pb ages: The Makalu case Himalaya. Earth. Planet. Sci. Lett..

[100] Rubatto D, Hermann J (2007). Experimental zircon/melt and zircon/garnet trace element partitioning and implications for the geochronology of crustal rocks. Chem. Geol..

[101] Smoliar MI, Walker RJ, Morgan JW (1996). Re-Os ages of group IIA, IIIA, IVA, and IVB iron meteorites. Science.

[102] Selby D, Creaser RA, Stein HJ, Markey RJ, Hannah JL (2007). Assessment of the 187Re decay constant by cross calibration of Re–Os molybdenite and U-Pb zircon chronometers in magmatic ore systems. Geochim. Cosmochim. Acta..

[103] Schoene B, Latkoczy C, Schaltegger U, Günther D (2010). A new method integrating high-precision U-Pb geochronology with zircon trace element analysis (U-Pb TIMS-TEA). Geochim. Cosmochim. Acta..

[104] Nowell, G. M. & Parrish, R. R. Simultaneous acquisition of isotope compositions and parent/daughter ratios by non-isotope dilution-mode plasma ionisation multi-collector mass spectrometry (PIMMS). In Plasma Source Mass Spectrometry: The New Millennium (Holland, G. & Tanner, S.D. eds) *Royal Soc. Chem.,Spec. Publ.* **267** 298–310 (2001).

[105] Lawley CJM, Selby D (2012). Re-Os geochronology of quartz enclosed ultra-fine Molybdenite: Implications for ore geochronology. Econ. Geol..

[106] Selby D, Creaser RA (2004). Macroscale NTIMS and microscale LA-MC-ICP-MS Re-Os isotopic analysis of molybdenite: Testing spatial restrictions for reliable Re-Os age determinations, and implications for the decoupling of Re and Os within molybdenite. Geochim. Cosmochim. Acta..

[107] Gramlich JW, Murphy TJ, Garner EL, Shields WR (1973). Absolute isotopic abundance ratio and atomic weight of a reference sample of rhenium. J. Res. Nat. Bur. Stand..

[108] Markey R, Stein HJ, Hannah JL, Zimmerman A, Selby D, Creaser RA (2007). Standardizing Re-Os geochronology: A new molybdenite reference material (Henderson, USA) and the stoichiometry of Os salts. Chem. Geol..

[109] Zimmerman A, Stein HJ, Morgan JW, Markey RJ, Watanabe Y (2014). Re-Os geochronology of the El Salvador porphyry Cu-Mo deposit, Chile: Tracking analytical improvements in accuracy and precision over the past decade. Geochim. Cosmochim. Acta..

[110] Hudson, D. M. & Oriel, W. M. (1979) Geologic map of the buckskin range. Nevada Bureau of Mines and Geology map 64 1

[111] McDonough WF, Sun SS (1995). The composition of the Earth. Chem. Geol..

[112] Middlemost EAK (1994). Naming materials in the magma/igneous rock system. Earth Sci. Rev..

