# Supplementary material for: A rapid change in magma plumbing taps porphyry copper deposit-forming magmas
Source: Sci Rep. 2022 Oct 14;12:17272. doi: 10.1038/s41598-022-20158-y (PMC9568598; doi:10.1038/s41598-022-20158-y)
Supplement: Supplementary file 7 — Supplementary Information 7. [file 41598_2022_20158_MOESM7_ESM.pdf]

Supplementary figures 1-18

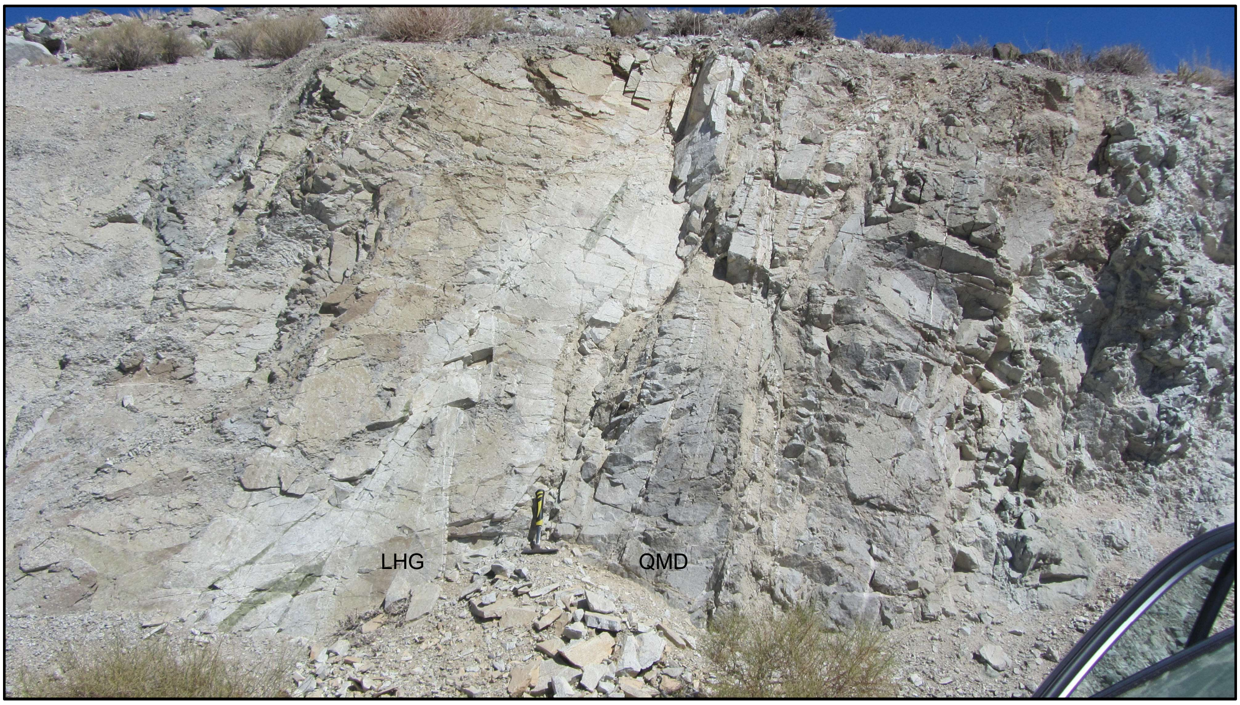

Figure S1. Plutonic contacts: Field photograph of the sharp contact between the McLeod QMD and LHG in the Luhr Hill area. Hammer placed at contact. No chilled margins or evidence of interaction is apparent. No metasomatic effects are present at the contacts beyond the later, pervasive, mostly Na-Ca and propylitic hydrothermal alteration.

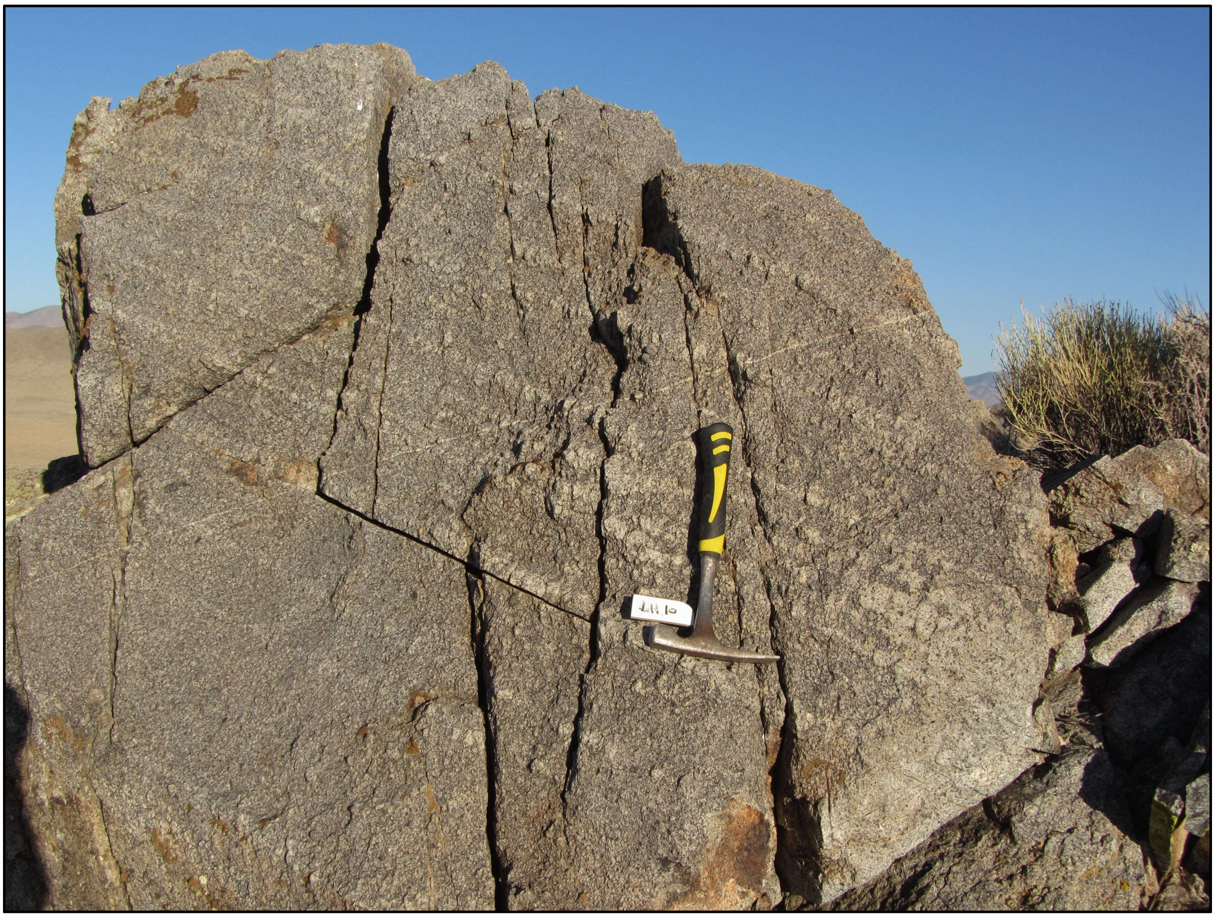

Figure S2. Igneous banding in Luhr Hill granite: Field photograph from a ~7.5 km palaeo-deep portion[27] of the LHG (Luhr Hill area) showing igneous banding, defined by grain size variations. We interpret this texture to be the product of magmatic differentiation processes post emplacement of the granitic melts into the upper crust.

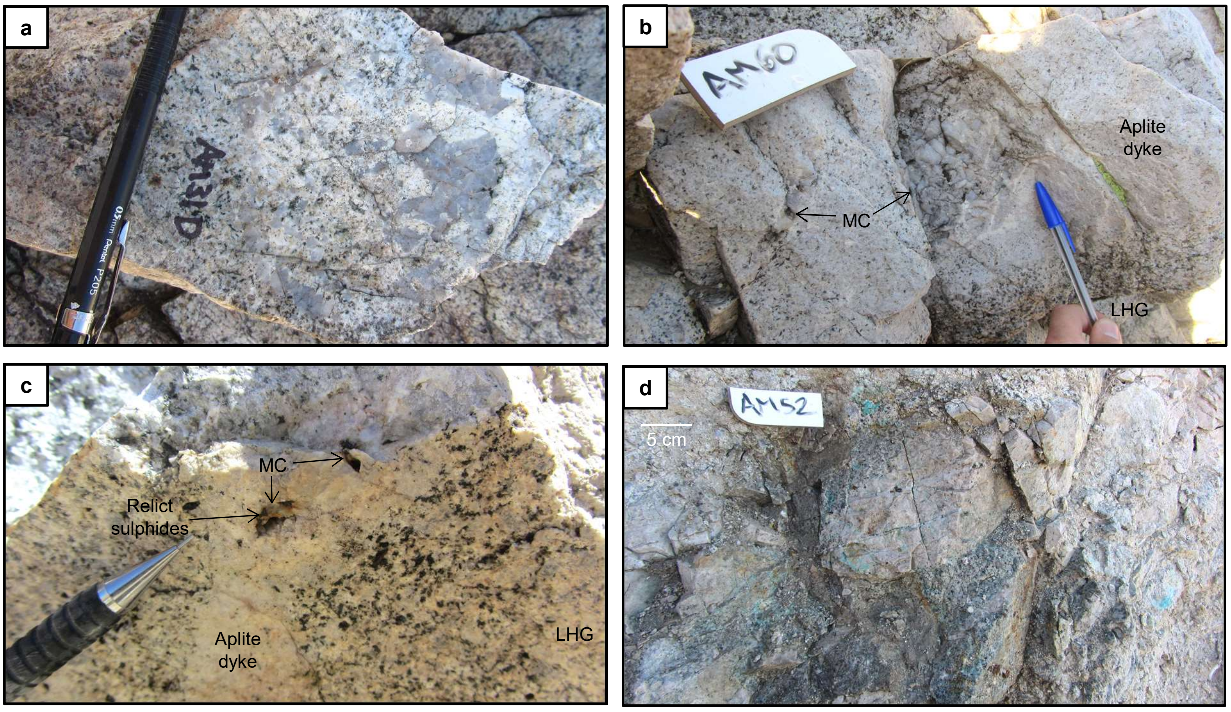

Figure S3. Textural evidence for undercooling, magmatic-hydrothermal fluid exsolution and mineralisation in aplite dykes: Field photographs showing; **a,** Quartz unidirectional solidification textures (USTs) in an aplite dyke which cuts the LHG cupola palaeo-vertically beneath the Ann Mason porphyry deposit; **b & c,** cupola zone of LHG cut by an aplite dyke which hosts mineralised miarolitic cavities (MC); **d,** Mineralised aplite dyke cutting the LHG cupola bearing chalcopyrite-mineralised A- and B-type quartz veins (nomenclature after [51]). Secondary copper staining prevalent.

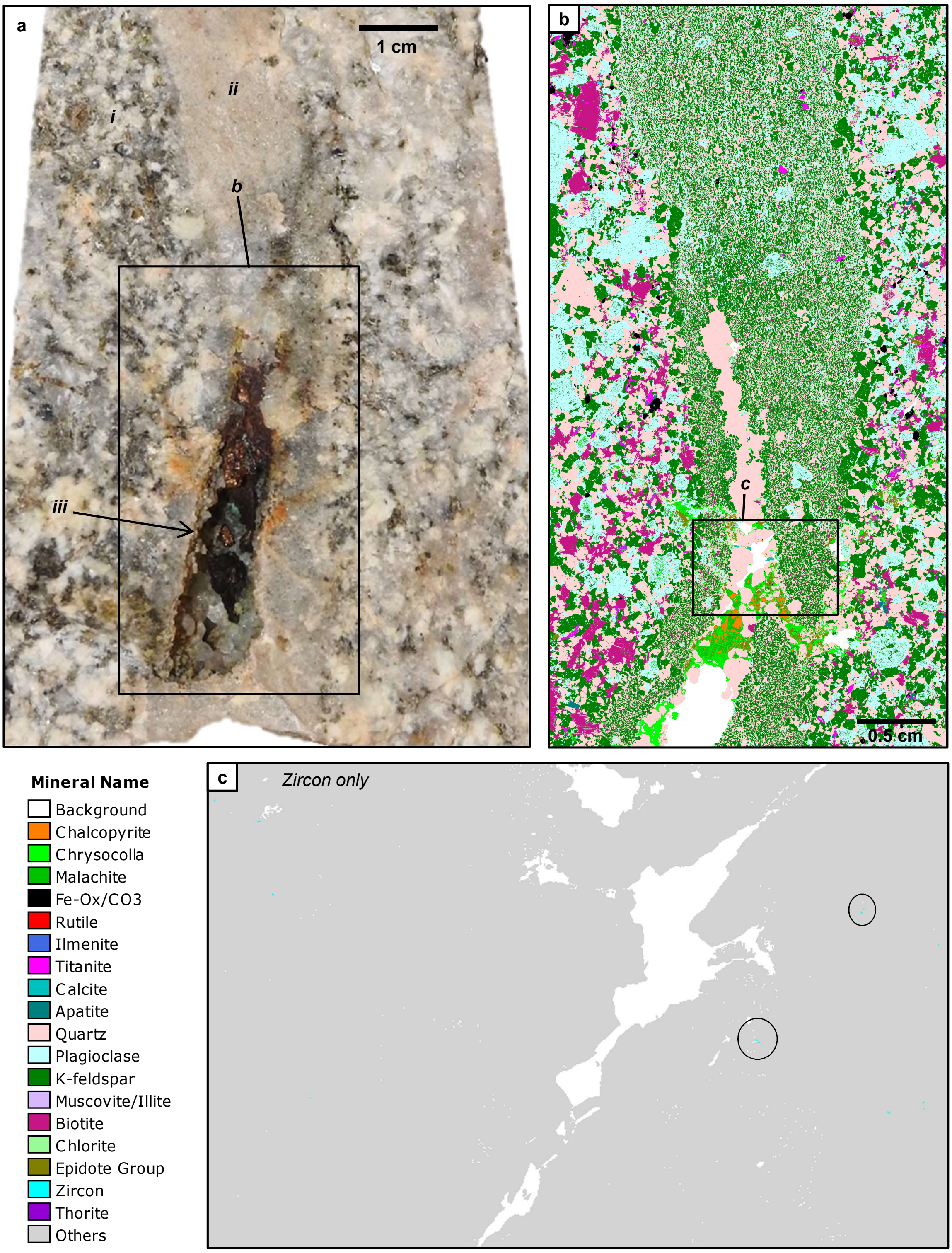

Figure S4. Zircons in aplite dyke which hosts mineralised miarolitic cavities: a, Photograph showing LHG from the cupola zone *(i)* cut by an aplite dyke *(ii)* which hosts hypogene Cu-mineralised miarolitic cavities *(iii)*. Box shows location of **b**; **b,** QEMSCAN mineral map of all phases. Box shows location of **c**; **c,** QEMSCAN map of only zircon (circled), seen at the margins of the miarolitic cavity within the aplite dyke. This aplite dyke is sample AM13BAP presented in Fig. 4. **A & b** from [33].

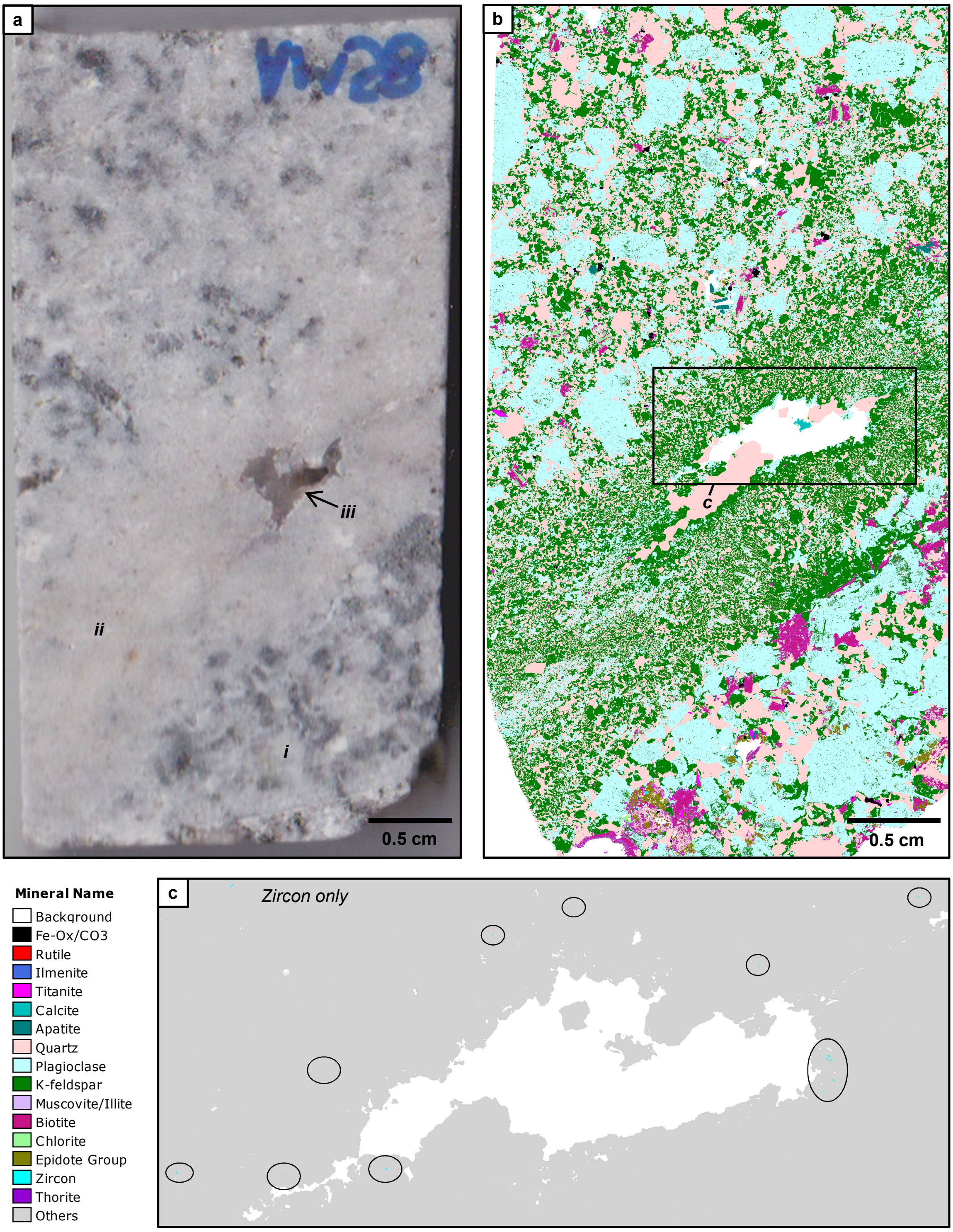

Figure S5. Zircons in aplite dyke which hosts miarolitic cavities: a, Photograph of rock chip showing LHG from the cupola zone *(i)* cut by an aplite dyke *(ii)* which hosts miarolitic cavities *(iii)*; **b,** QEMSCAN mineral map of all phases, same field of view as **a**. Box shows location of **c**; **c,** QEMSCAN map of only zircon (circled), seen at the margins of the miarolitic cavity within the aplite dyke.

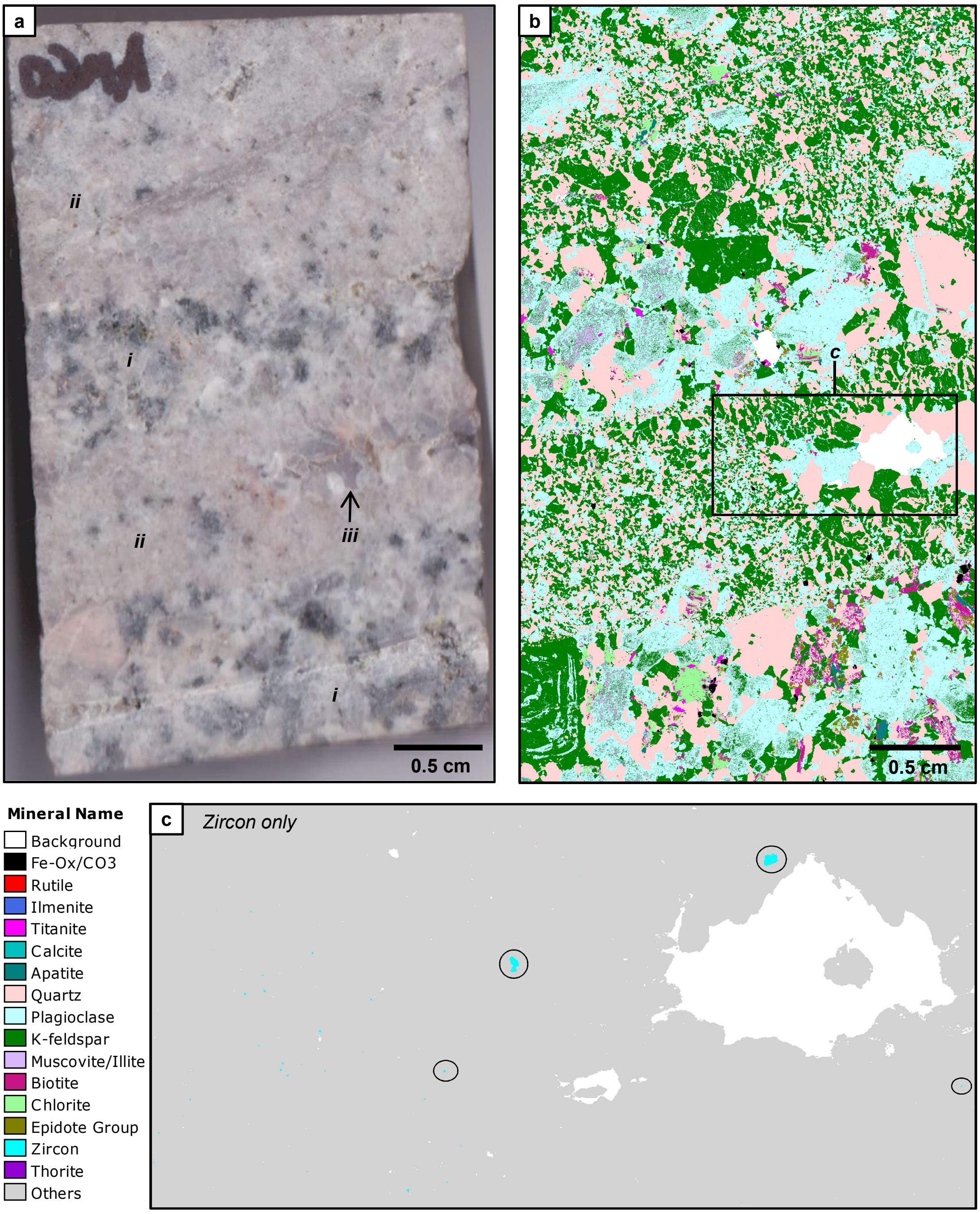

Figure S6. Zircons in aplite dyke which hosts miarolitic cavities: a, Photograph of rock chip showing LHG from the cupola zone *(i)* cut by two aplite dykes *(ii)* which host miarolitic cavities *(iii)*; **b,** QEMSCAN mineral map of all phases, same field of view as **a**. Box shows location of **c**; **c,** QEMSCAN map of only zircon (circled), seen at the margins of the miarolitic cavity within the aplite dyke.

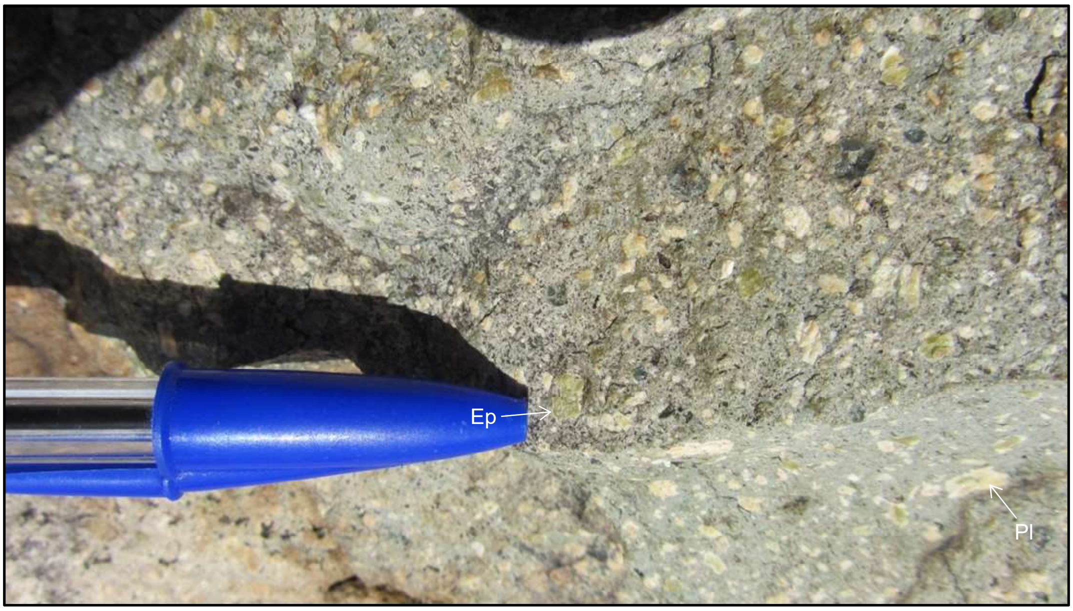

Figure S7. Propylitic alteration in Fulstone Spring volcanics: which overlie the Yerington porphyry system[27]. Epidote (apple green; Ep) is seen replacing primary plagioclase (milky white; Pl). Pen for scale. Zircon LA-ICP-MS trace element data for this sample (BS10) is presented in Fig. 7.

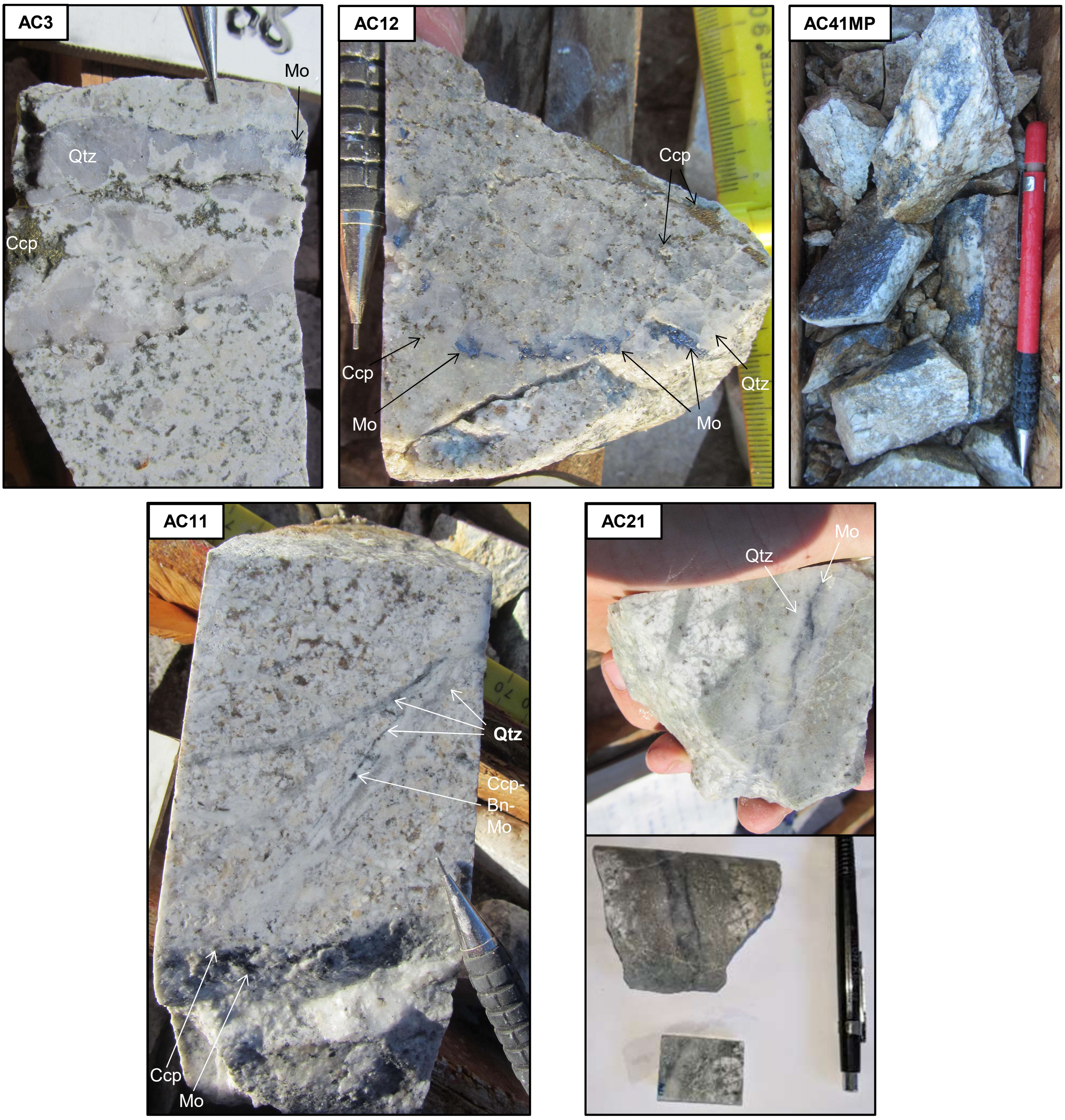

Figure S8. Samples for molybdenite Re-Os age determinations: Photographs of drill core samples from the Ann Mason porphyry copper deposit. Sample AC3 is a chalcopyrite-molybdenite (Ccp-Mo) bearing quartz (Qtz) UST within an aplite dyke (or vein-dyke texture). Sample AC12 is a chalcopyrite-molybdenite-quartz vein cutting LHG. Sample AC41MP is a fine grained molybdenite vein cutting LHG which was approximately split into 4 equal subsamples. This splitting into subsamples provides the opportunity to check the consistency and closed behaviour of the Re-Os system within the vein. Sample AC11 is of sinuous (A-type) chalcopyrite-bornite(Bn)-molybdenite-quartz veins cutting an aplite dyke and LHG. Sample AC21 of is a planar (B-type) chalcopyrite-molybdenite-quartz vein with ~1 cm K-feldspar halo, with fine grained molybdenite in the central suture. See Supplementary Data 1 for further details.

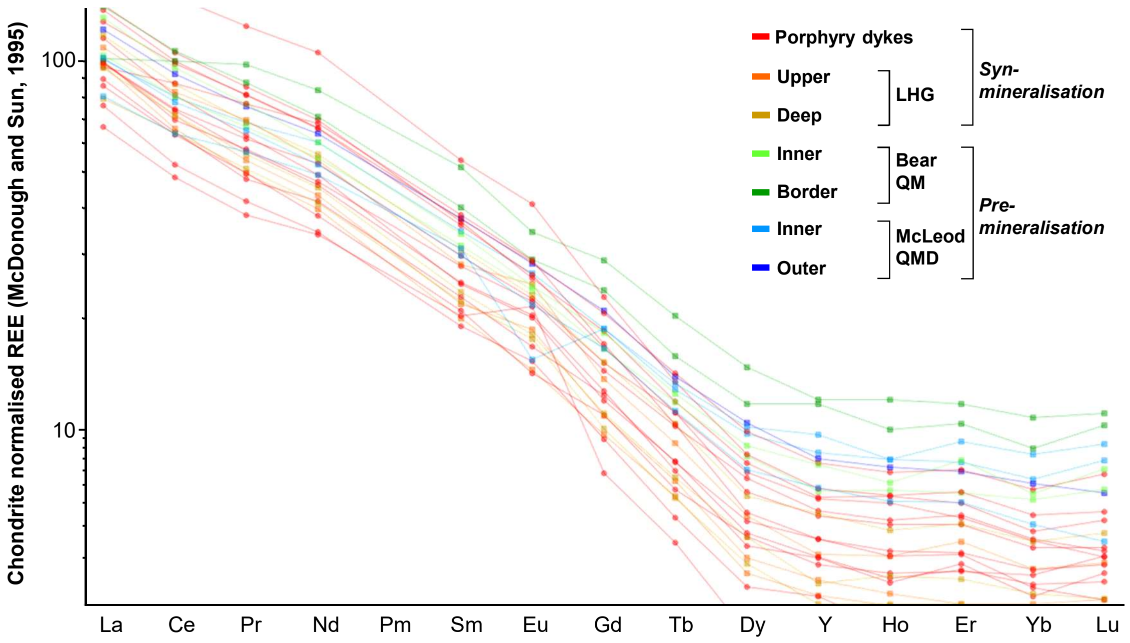

Figure S9. Chondrite-normalized[111] mean whole-rock REE plots.

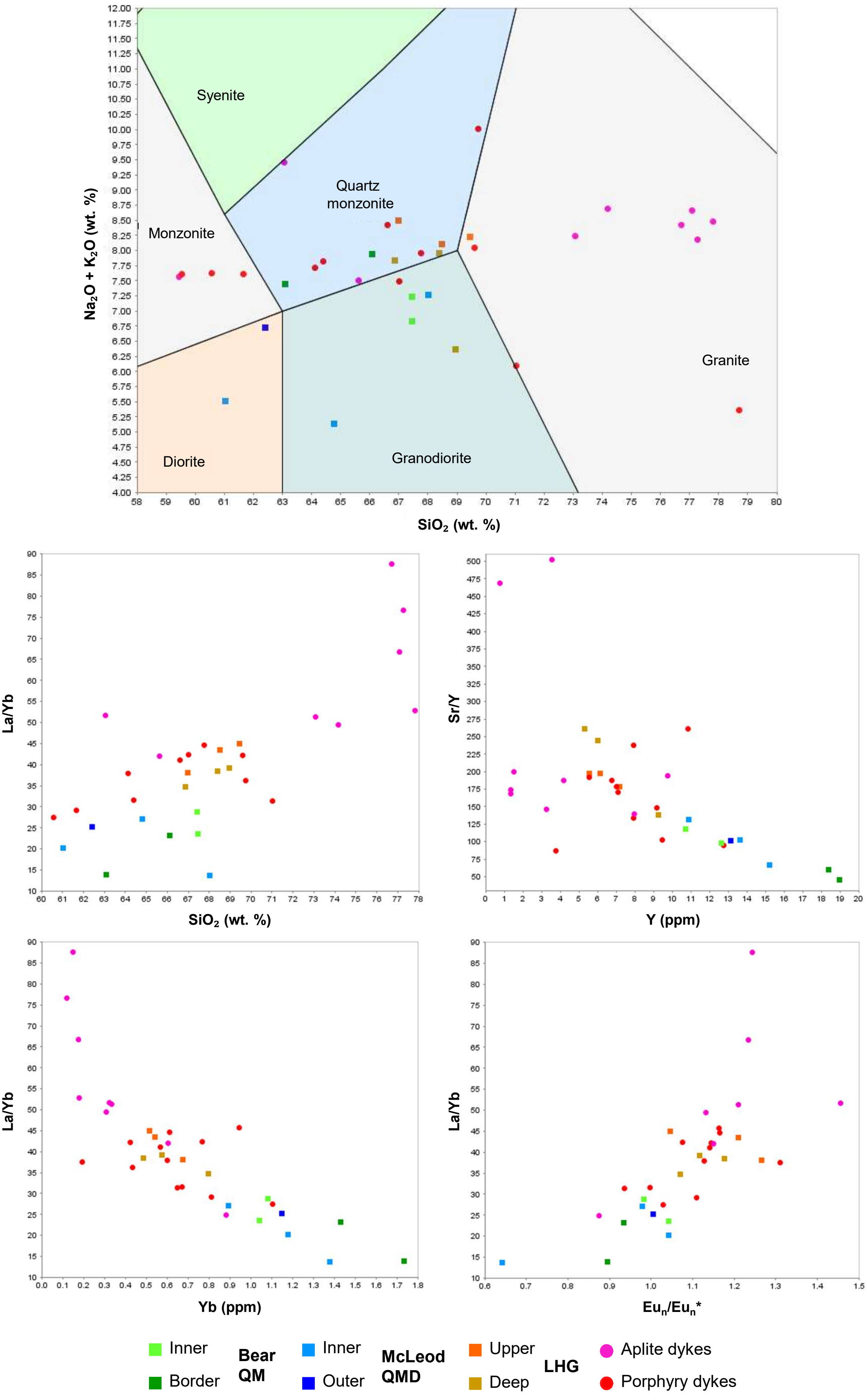

Figure S10. Plutonic TAS diagram[112] and whole-rock geochemistry through the Yerington magmatic system. Major elements partially overlap between the mineralogically distinct[27] intrusive units. Distinct differences between pre- and syn-mineralisation units are seen in trace element ratios.

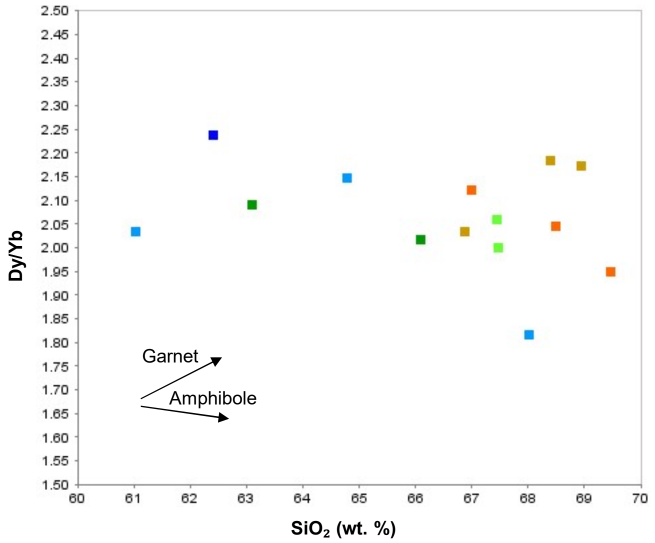

Figure S11. Dy/Yb versus SiO_2 for plutonic units. Whilst Dy/Yb values remain ~2, a slightly negative trend is seen with increasing SiO_2 content, following the amphibole fractionation trend and suggesting that garnet did not play a role in the geochemical evolution of the system. Vectors for garnet and amphibole fractionation from [64] .

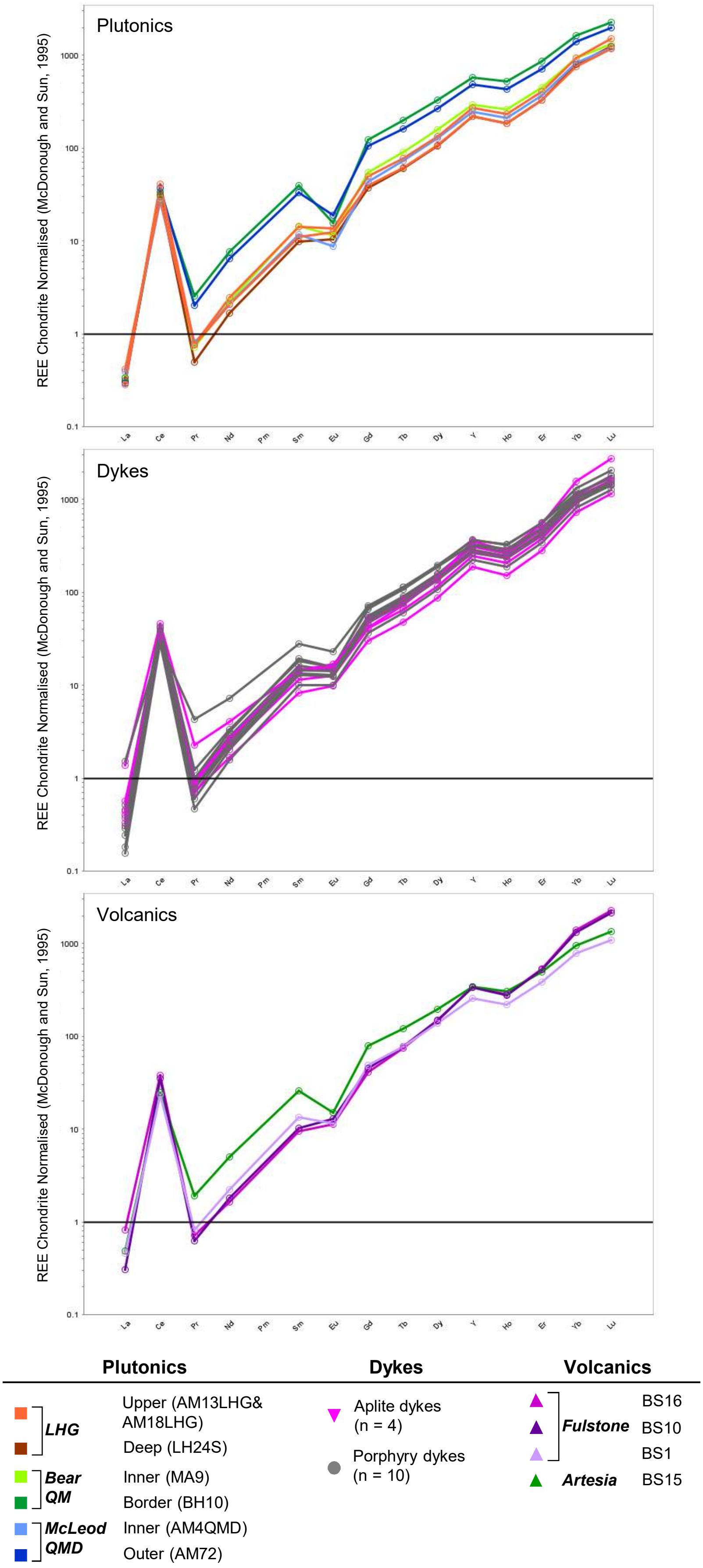

Figure S12. Zircon REE patterns: Zircon LA-ICP-MS chondrite normalised[111] REE patterns from samples temporally and spatially spanning the Yerington magmatic system.

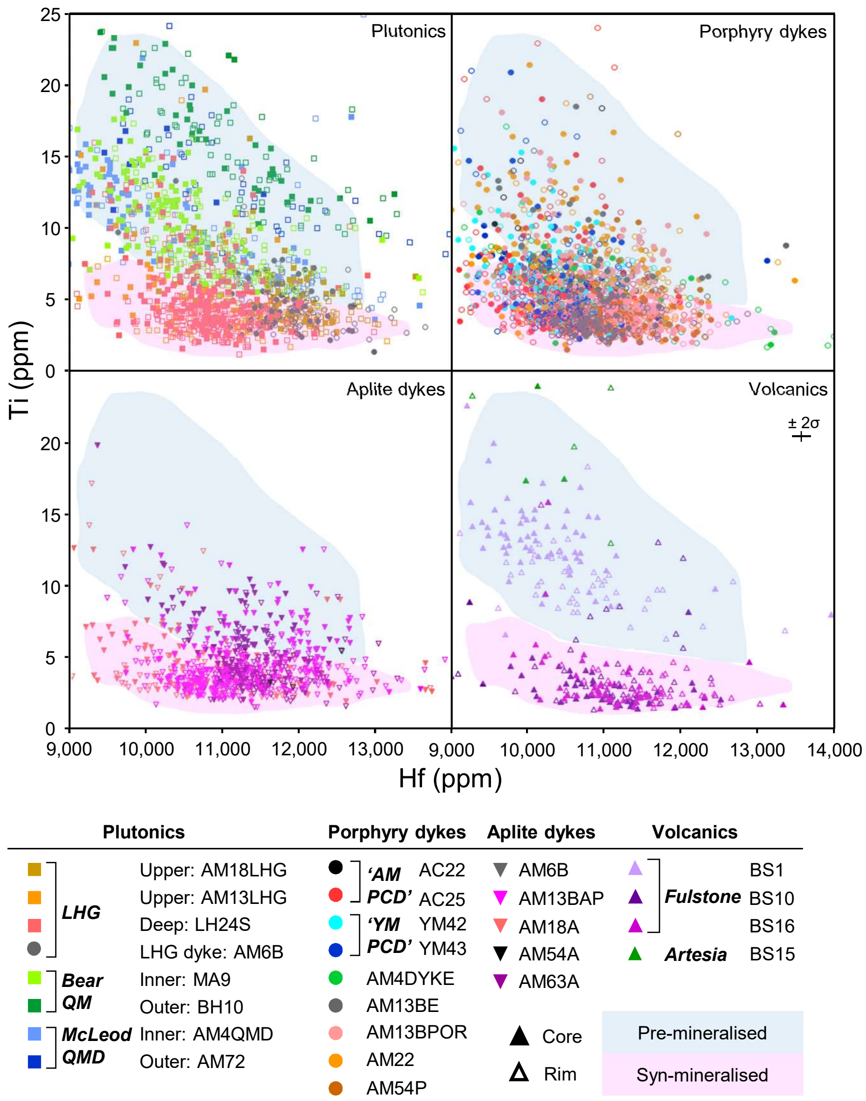

Figure S13. Zircon trace element signatures through the Yerington magmatic system: Zircon LA-ICP-MS trace element data from samples spanning, temporally and spatially, the Yerington magmatic system. Both core and rim data plotted. 'Pre-mineralised' and 'syn-mineralised' fields shaded. AM = Ann Mason porphyry deposit. YM = Yerington porphyry deposit.

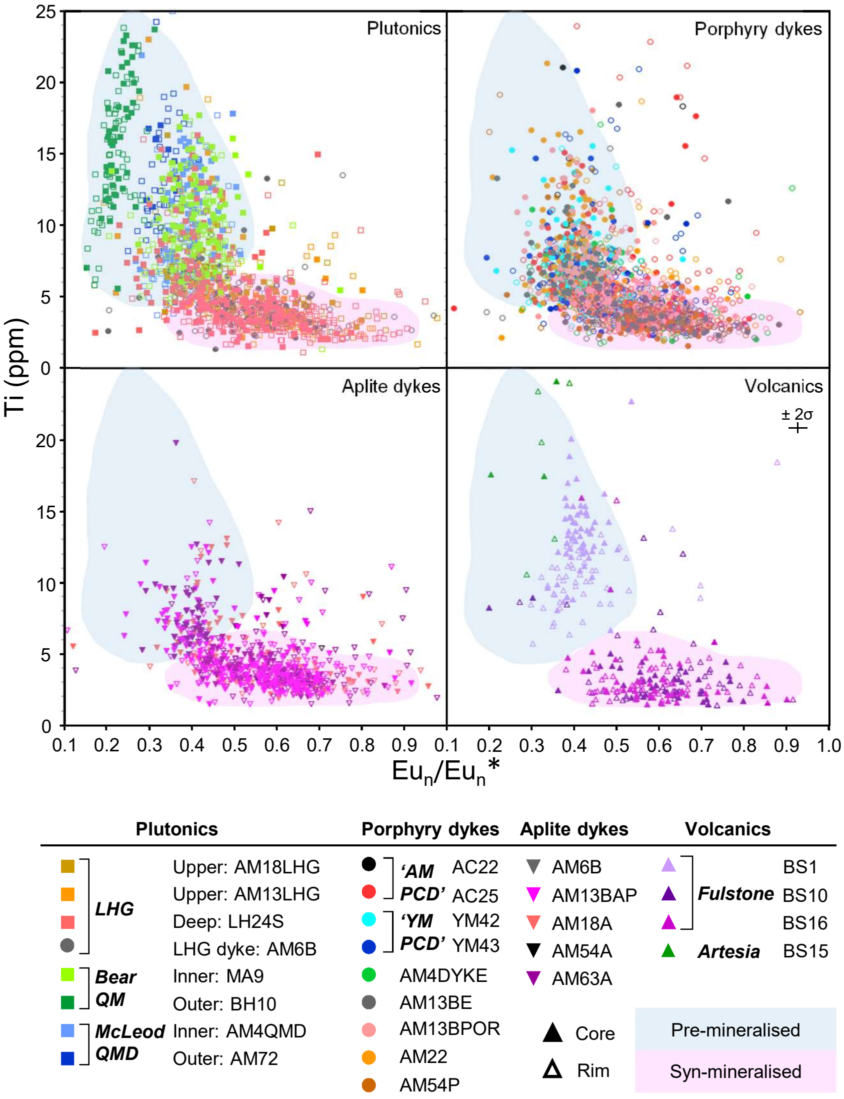

Figure S14. Zircon trace element signatures through the Yerington magmatic system: Zircon LA-ICP-MS trace element data from samples spanning, temporally and spatially, the Yerington magmatic system. Both core and rim data plotted. 'Pre-mineralised' and 'syn-mineralised' fields shaded. AM = Ann Mason porphyry deposit. YM = Yerington porphyry deposit.

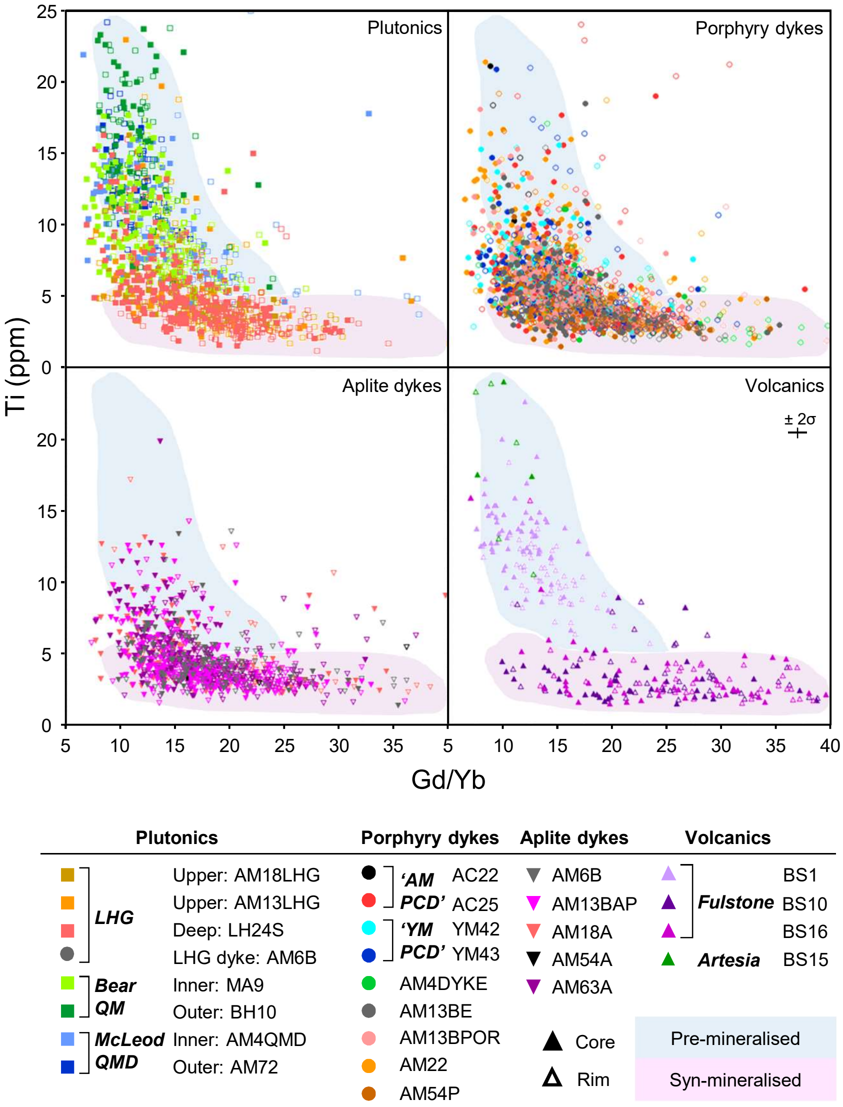

Figure S15. Zircon trace element signatures through the Yerington magmatic system: Zircon LA-ICP-MS trace element data from samples spanning, temporally and spatially, the Yerington magmatic system. Both core and rim data plotted. 'Pre-mineralised' and 'syn-mineralised' fields shaded. AM = Ann Mason porphyry deposit. YM = Yerington porphyry deposit.

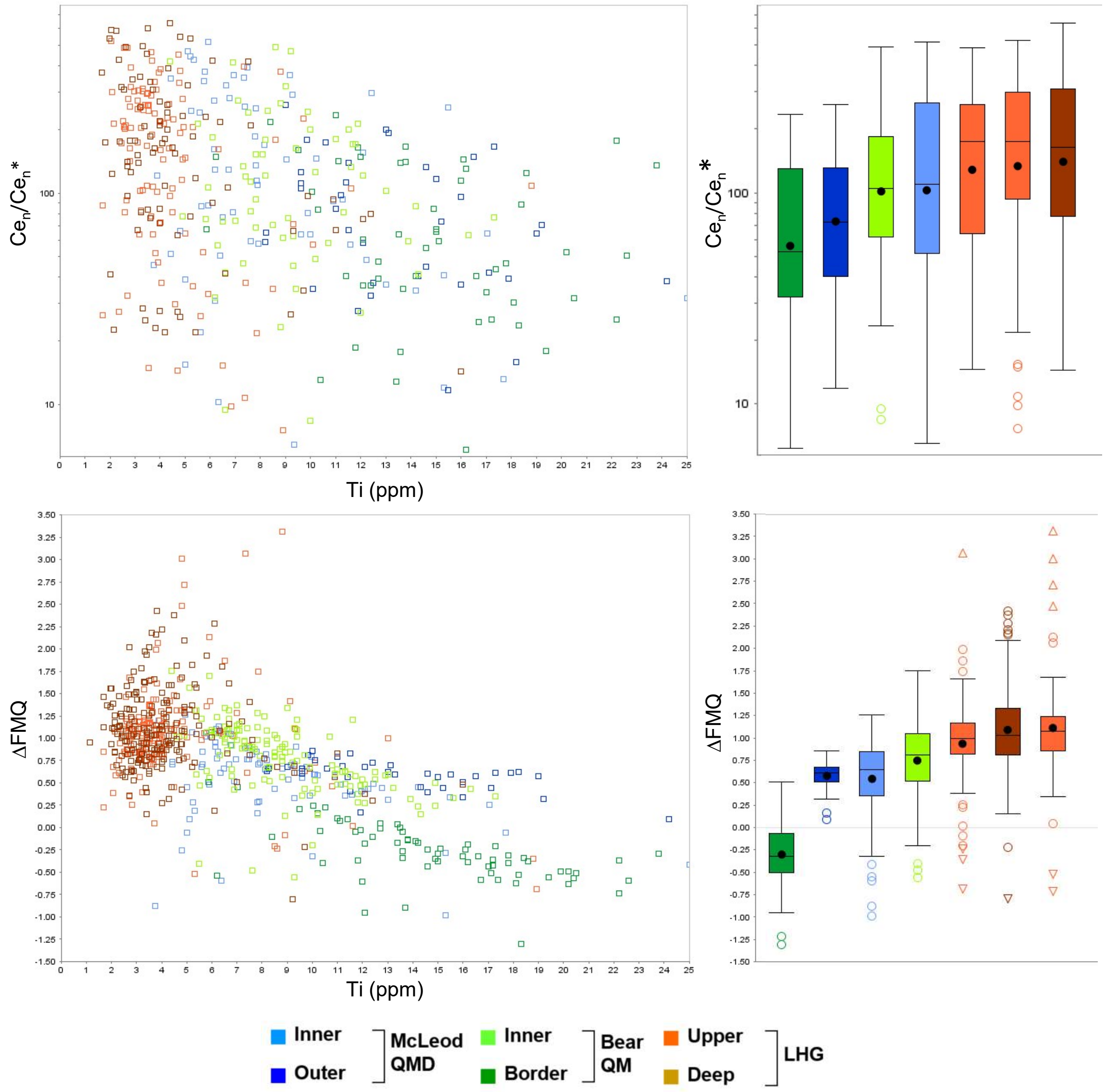

Figure S16. Zircon trace element signatures for plutonic units of the Yerington porphyry system: Ce/Ce* versus Ti; Ce/Ce* box and whisker plot; ΔFMQ versus Ti; ΔFMQ box and whisker plot. Ce/Ce* calculated using method of [70]. ΔFMQ calculated using method of [69]. Only zircon rim data plotted.

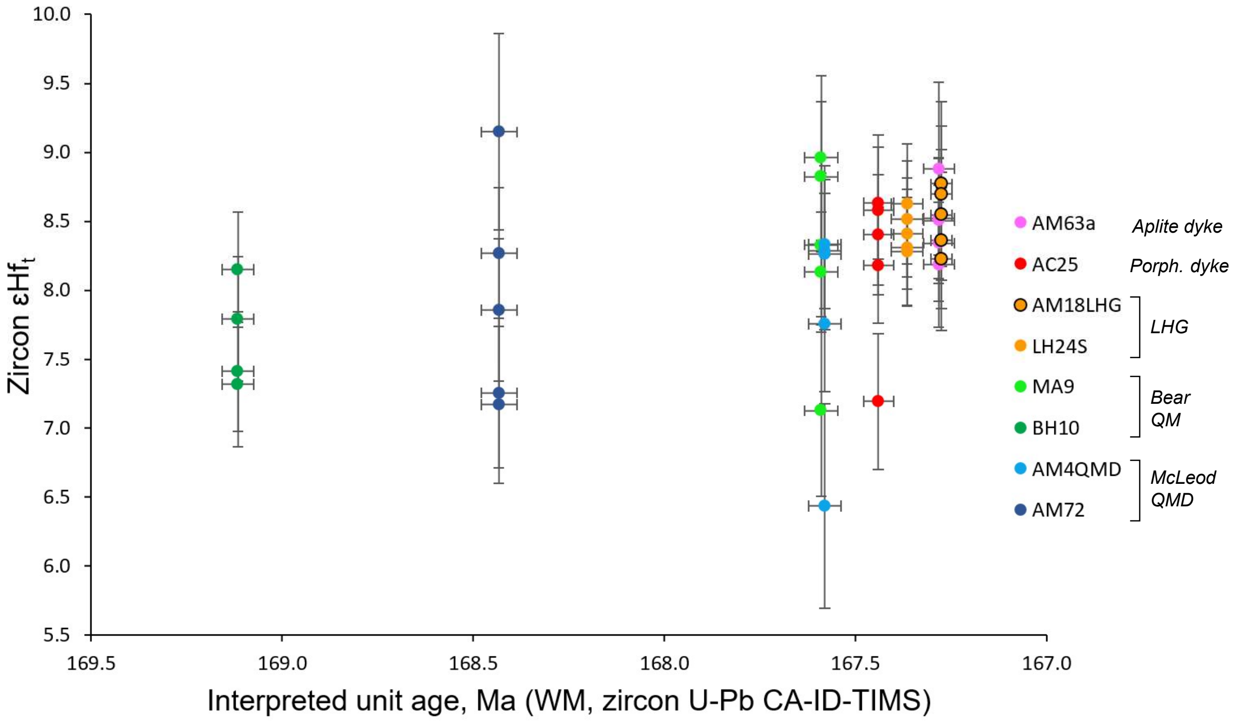

Figure S17. Zircon εHf$_t$ through the Yerington magmatic system: Time corrected zircon εHf (εHf$_t$) versus interpreted zircon age for samples spanning the Yerington magmatic system. Age determinations for each sample are weighted mean (WM) from zircon single grain U-Pb CA-ID-TIMS analyses, with error bars at 2σ (Fig. 4). εHf$_t$ error bars 2σ.

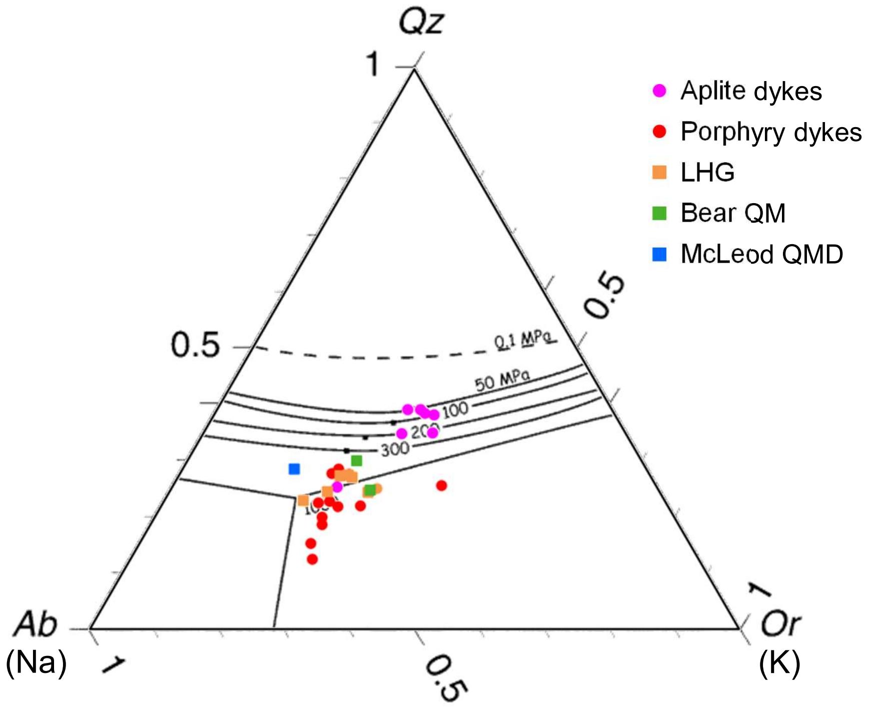

Figure S18. Depth of different magma sources: CIPW normative mineralogy (method [90]) from whole-rock XRF data plotted on the H_2O-saturated haplogranitic melt minima plot from [78]. Cotectic lines and eutectics are a function of pressure and therefore the whole-rock data can be used to provide constraints for the pressure of magma differentiation[67], from which depth can be approximated. Porphyry dyke samples are plotted here in addition to the McLeod QMD, Bear QM, LHG and aplite dyke samples plotted in Fig. 9. Porphyry dykes appear to plot along with the LHG samples around the ~450-1000 MPa minima. Samples overprinted by pervasive Na-Ca alteration are not plotted.
